# Historical museum collections clarify the evolutionary history of cryptic species radiation in the world's largest amphibians

**DOI:** 10.1002/ece3.5257

**Published:** 2019-09-16

**Authors:** Samuel T. Turvey, Melissa M. Marr, Ian Barnes, Selina Brace, Benjamin Tapley, Robert W. Murphy, Ermi Zhao, Andrew A. Cunningham

**Affiliations:** ^1^ Institute of Zoology Zoological Society of London London UK; ^2^ Earth Sciences Department Natural History Museum London UK; ^3^ Living Collections Zoological Society of London London UK; ^4^ Centre for Biodiversity and Conservation Biology Royal Ontario Museum Toronto Ontario Canada; ^5^ State Key Laboratory of Genetic Resources and Evolution, Kunming Institute of Zoology Chinese Academy of Sciences Kunming China; ^6^ Chengdu Institute of Biology Chinese Academy of Sciences Chengdu China

**Keywords:** amphibian, *Andrias*, Chinese giant salamander, conservation, cryptic species, historical baselines, translocation

## Abstract

Inaccurate taxonomic assessment of threatened populations can hinder conservation prioritization and management, with human‐mediated population movements obscuring biogeographic patterns and confounding reconstructions of evolutionary history. Giant salamanders were formerly distributed widely across China, and are interpreted as a single species, *Andrias davidianus*. Previous phylogenetic studies have identified distinct Chinese giant salamander lineages but were unable to associate these consistently with different landscapes, probably because population structure has been modified by human‐mediated translocations for recent commercial farming. We investigated the evolutionary history and relationships of allopatric Chinese giant salamander populations with Next‐Generation Sequencing methods, using historical museum specimens and late 20th‐century samples, and retrieved partial or near‐complete mitogenomes for 17 individuals. Samples from populations unlikely to have been affected by translocations form three clades from separate regions of China, spatially congruent with isolation by either major river drainages or mountain ranges. Pliocene–Pleistocene divergences for these clades are consistent with topographic modification of southern China associated with uplift of the Qinghai‐Tibet Plateau. General Mixed Yule Coalescent model analysis indicates that these clades represent separate species: *Andrias davidianus* (Blanchard, 1871) (northern Yangtze/Sichuan), *Andrias sligoi* (Boulenger, 1924) (Pearl/Nanling), and an undescribed species (Huangshan). *Andrias sligoi* is possibly the world's largest amphibian. Inclusion of additional reportedly wild samples from areas of known giant salamander exploitation and movement leads to increasing loss of biogeographic signal. Wild Chinese giant salamander populations are now critically depleted or extirpated, and conservation actions should be updated to recognize the existence of multiple species.

## Graphical abstract

Giant salamanders were formerly distributed widely across China, and are interpreted as a single species, *Andrias davidianus*. We investigated the evolutionary history and relationships of allopatric Chinese giant salamander populations with Next‐Generation Sequencing methods, using historical museum specimens and late 20th century samples, and retrieved partial or near‐complete mitogenomes for 17 individuals. Samples cluster within three clades from separate regions of China, and represent three separate species, of which *Andrias sligoi* rather than *Andrias davidianus* is possibly the world's largest amphibian.

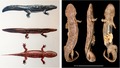

## 1 INTRODUCTION

The conservation of highly threatened taxa is dependent upon the availability of robust baseline information on key population parameters (Segan, Bottrill, Baxter, & Possingham, 2011; Sutherland, Pullin, Dolman, & Knight, 2004). Most fundamentally, determining species boundaries and understanding the taxonomic identity and distinctiveness of populations of conservation concern are essential steps needed to define appropriate management units and inform effective management decisions (Mace, 2004; May, 1990; Tapley et al., 2018). However, many recent examples exist of conservation efforts being delayed or misdirected due to inaccurate taxonomic assessment, in some cases resulting in catastrophic decline or even extinction of evolutionarily significant populations and distinct species (e.g., Beebee et al., 2005; Iglésias, Toulhoat, & Sellos, 2010; Robertson, Stephenson, & Goldstien, 2011). Increased assessment of taxonomic status for threatened populations is therefore urgently required to guide biodiversity conservation (Costello, Vanhoorne, & Appeltans, 2015; Thomson et al., 2018).

Global biodiversity has experienced extensive historical modification by human activities, which can hinder assessment of the taxonomic status of many populations. In particular, human‐mediated translocations can obscure biogeographic patterns, confound reconstructions of evolutionary history (Gippoliti & Amori, 2006; Helgen & Wilson, 2003), and remove spatial barriers to gene flow, promoting hybridization between taxa that were once geographically isolated (Fitzpatrick et al., 2010; Ladle & Whittaker, 2011). Conservation researchers and managers therefore increasingly make use of environmental archives (Bonebrake, Christensen, Boggs, & Ehrlich, 2010; Davies, Colombo, & Hanley, 2014), such as historical museum collections (Díez‐Del‐Molino, Sánchez‐Barreiro, Barnes, Gilbert, & Dalén, 2018; Hekkala et al., 2011; Turvey, Barnes, Marr, & Brace, 2017), to reconstruct past environmental baselines in systems that have experienced human modification of biodiversity, and to obtain novel insights into the evolution, ecology, and biogeography of species that have undergone historical range modifications. Understanding historical baselines and the extent to which human activities have disrupted biodiversity is of particular importance for regions with long histories of human modification that are now experiencing extreme anthropogenic pressure, notably ecosystems in eastern and southeast Asia (Marks, 2017; Turvey, Crees, Li, Bielby, & Yuan, 2017).

**Figure 1:**
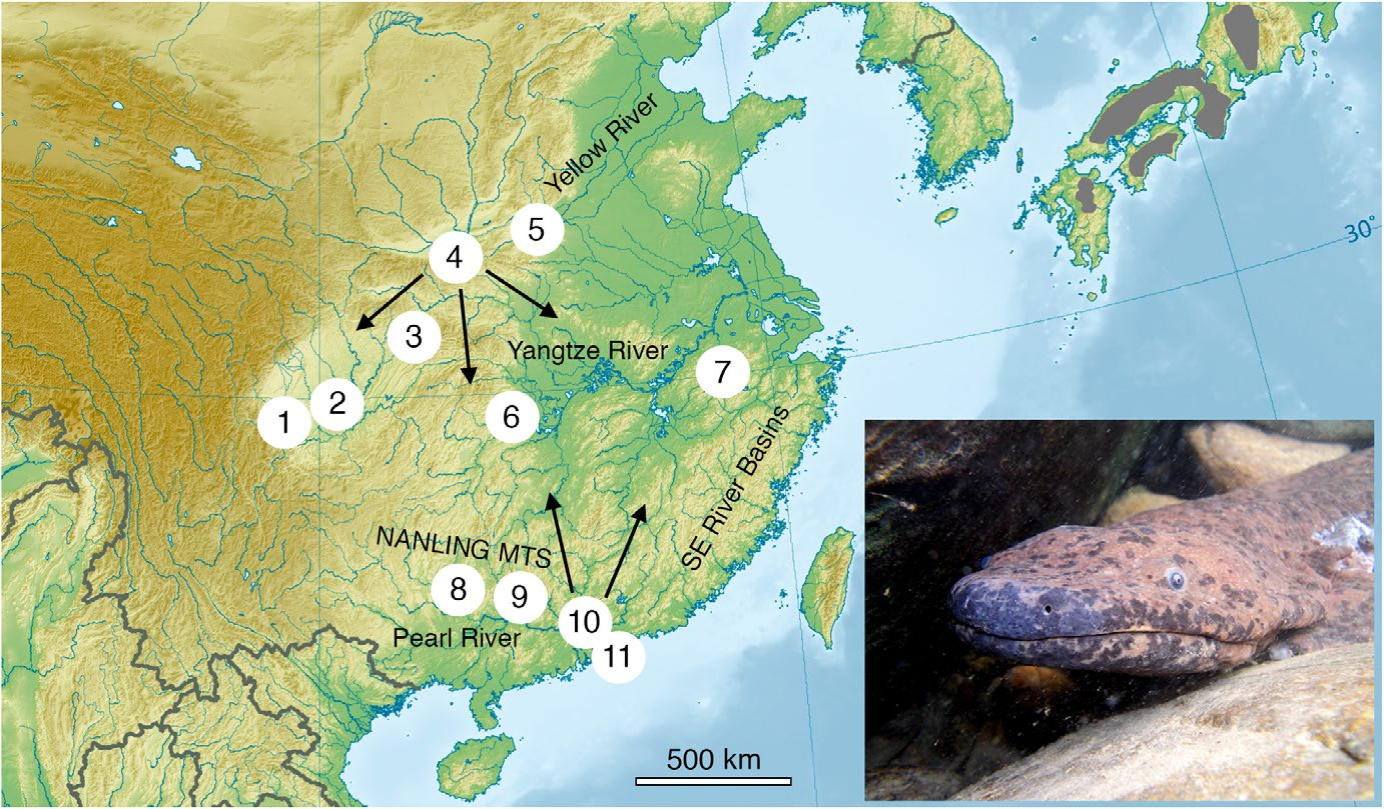
Map of eastern Asia showing Chinese river drainages and mountain regions, and giant salamander sample localities: 1, Ya'an; 2, Meishan; 3, Zhongba/Chongqing; 4, Xi'an; 5, Yuanqu; 6, Zhangjiajie; 7, Huangshan; 8, northern Guangxi; 9, northern Guangdong; 10, Guangzhou; 11, Hong Kong. Arrows indicate direction of human‐mediated movement of giant salamanders associated with trade and farming. Gray hatching indicates distribution of giant salamanders in Japan based on IUCN (2018). Inset, Chinese giant salamander (*Andrias davidianus*) found during 2013–2016 survey, assigned to clade B of Yan, Lü, et al. (2018); see Turvey et al., 2018

The Chinese giant salamander (*Andrias davidianus*), the world's largest amphibian, is a cryptobranchid salamander endemic to China, where it is a top predator in freshwater ecosystems (Fei, Hu, Ye, & Huang, 2006). Giant salamanders were formerly distributed across a large area of central, eastern, and southern China (Figure 1) and are recorded from 18 Chinese provinces or equivalent administrative regions (Chen et al., 2018; Fei et al., 2006). They were historically eaten and used for traditional medicine in parts of southern China (Simoons, 1991), and were sold for food from the historical trading center of Guangzhou (Canton) to cities such as Shanghai (Liu, 1950; Sowerby, 1925b), but were avoided and considered unlucky in other parts of their range (Cunningham et al., 2016). However, exploitation of giant salamanders increased in China from the late 1970s onwards, following the freeing of internal movement of people within China and the spread of southern Chinese migrants who had traditionally eaten the species; this led to trade and movement of animals across the country to supply a domestic luxury food market, and to development of a massive‐scale farming industry which has grown rapidly and expanded from southern Shaanxi Province across China since the early 2000s (Cunningham et al., 2016). The farming industry poses a huge threat to wild populations through continuing illegal harvesting of wild animals to stock farms, and serious risk of infectious disease transmission and genetic pollution associated with accidental escapes or deliberate “conservation” releases of farmed animals that have been moved around the country (Cunningham et al., 2016; Turvey et al., 2018; Yan, Lü, et al., 2018). The Chinese giant salamander is now listed as Critically Endangered by IUCN (2018) and is recognized as a global conservation priority for maintaining evolutionary history (Isaac, Redding, Meredith, & Safi, 2012), because there are only two other living cryptobranchids (Japanese giant salamander, *Andrias japonicus*; hellbender, *Cryptobranchus alleganiensis*), which are both listed as Near Threatened by IUCN (2018). A recent multi‐year (2013–2016) range‐wide survey detected Chinese giant salamanders at only four out of 97 sites, revealing that wild populations are now critically depleted or extirpated across all surveyed areas of China (Turvey et al., 2018) and highlighting the urgent need to identify priority populations and landscapes for targeted conservation attention.

The wide historical distribution of giant salamanders across China (Figure 1) includes the Yellow, Yangtze, and Pearl river drainages, as well as other smaller isolated river systems such as the southeastern Fujian‐Zhejiang hills drainage originating in Huangshan (Yellow Mountains) in Anhui (Zheng et al., 2016). These separate drainages represent distinct biogeographic regions with independent geological histories and exhibit substantial endemism in freshwater taxa (Fei et al., 2006; Shih & Ng, 2011; Xing, Zhang, Fan, & Zhao, 2016; Zieritz et al., 2018). Giant salamanders are fully aquatic and occur in fast‐flowing tributaries within mountainous landscapes (Chen et al., 2018) across a series of different montane ecoregions that also represent areas of extensive local endemism (López‐Pujol, Zhang, Sun, Ying, & Ge, 2011a, 2011b; Tang, Wang, Zheng, & Fang, 2006), and that can act as watersheds for multiple river drainages (e.g., Nanling mountains separate the Yangtze and Pearl drainages; Huangshan separates the Yangtze and Fujian‐Zhejiang hills drainages). Previous molecular phylogenetic analyses of giant salamander samples collected from across China (Murphy, Fu, Upton, Lema, & Zhao, 2000; Tao, Wang, Zheng, & Fang, 2005; Wang, Zhang, Xie, Wei, & Jiang, 2017; Yan, Lü, et al., 2018), which were investigated using isozymes, mitochondrial genes, microsatellites, or single nucleotide polymorphisms (SNPs), have identified genetically distinct local populations (e.g., a genetically distinct population from Huangshan; Murphy et al., 2000; Yan, Lü, et al., 2018). The most recent genetic study of wild‐caught and farmed Chinese giant salamander samples identified seven distinct lineages using mitochondrial genes (partial cytochrome *b* [cyt*b*], *COI*, D‐loop) and nuclear SNPs (Yan, Lü, et al., 2018); some or all of these lineages were considered likely to represent cryptic species, thus revealing previously unsuspected levels of diversity within Chinese cryptobranchids. However, these studies did not detail their findings against regional biogeographic patterns shown by other Chinese taxa and were also unable to consistently associate distinct clades with different landscapes, which they attributed to recent human movement of giant salamanders around China modifying local population structure and obscuring historical patterns of regional biogeographic differentiation (Murphy et al., 2000; Yan, Lü, et al., 2018). Indeed, genetic analysis of individuals found in the Yangtze and Pearl drainages during the 2013–2016 survey revealed these individuals all possessed a matriline characteristic of farmed individuals that originated from the Yellow River drainage, strongly suggesting they were farm escapes/releases (Turvey et al., 2018). Current‐day samples are therefore unlikely to be able to reconstruct landscape‐level evolutionary history and biogeography of giant salamander populations across China.

**Figure 2:**
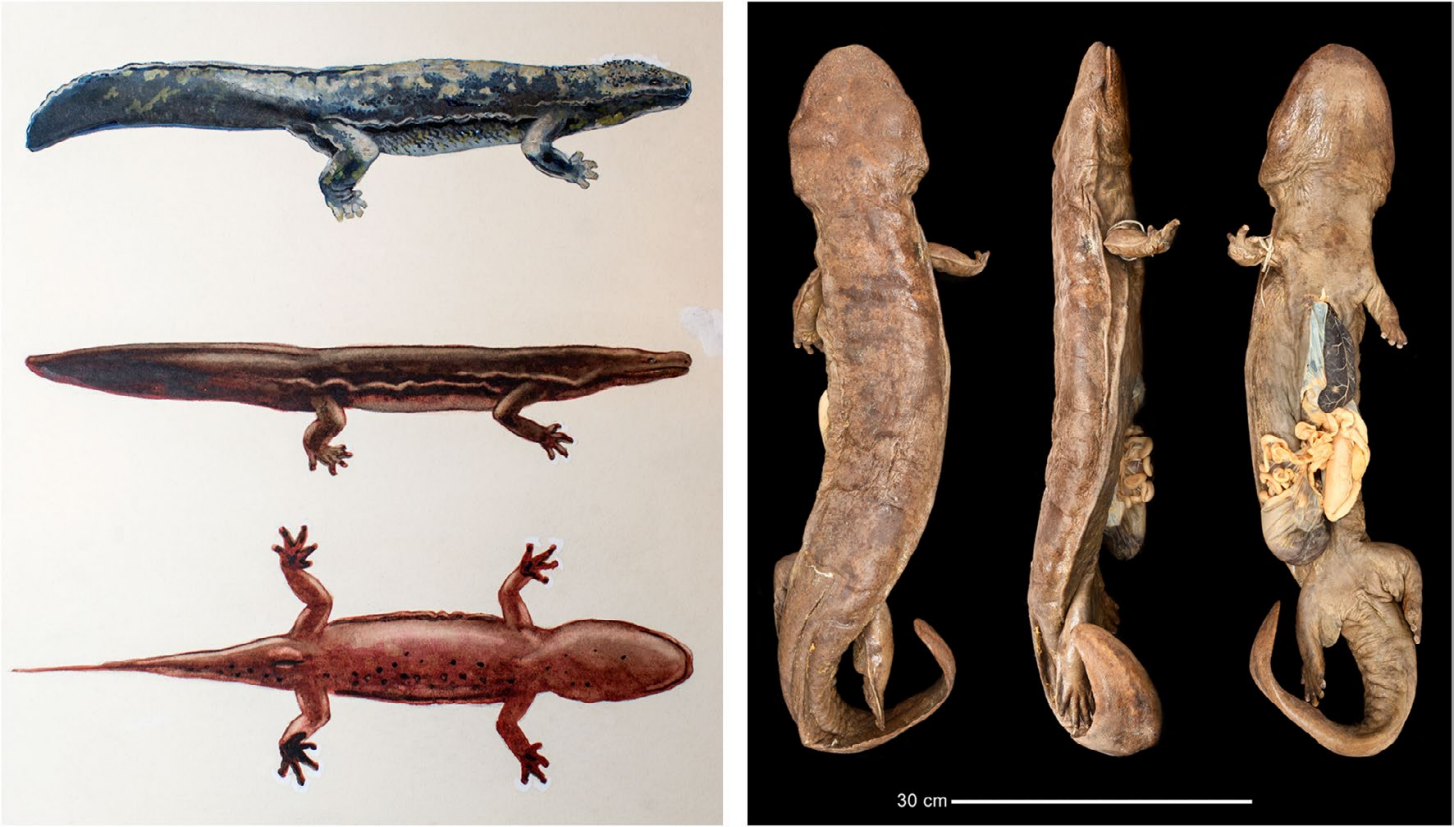
Left, previously unpublished painting showing *Andrias japonicus* (top) and *Andrias sligoi* (middle, bottom), probably originally prepared for inclusion in Boulenger (1924). Artist unknown; courtesy of Zoological Society of London library. Right, holotype of *A. sligoi* (BMNH 1945.11.7.1), dorsal, lateral, and ventral views

Whereas previous Chinese giant salamander molecular studies have relied upon samples collected in recent decades, numerous historical specimens of known provenance exist in museum collections (Table 1; Table S1). These specimens were obtained before giant salamanders began to be moved extensively around China in the late twentieth century (Cunningham et al., 2016) and so are likely to represent native local populations. They include a specimen found in the Hong Kong Botanical Gardens in 1920 and thought to have been brought from the nearby mainland (Guangdong or Guangxi provinces; Liu, 1950), which was described in 1924 as a separate species of giant salamander, *Megalobatrachus sligoi* (Boulenger, 1924); this putative taxon was subsequently synonymized with *Andrias davidianus* (Thorn, 1968) and has since been largely forgotten, but may represent one of the cryptic giant salamander “species lineages” identified from analysis of recent samples (Figure 2; Text S1 and Figure S1).

**Table 1:** Specimen information for the 17 Chinese giant salamander samples which successfully yielded partial or near‐complete mitogenomes. Analysis number indicates whether samples were included in analysis of populations unlikely to have been affected by translocations (Analysis 1), or only in analysis of all mitogenomic data (Analysis 2)

Specimen ID	Tissue type	Collection date	Locality	Drainage	x‐fold coverage	Proportion of mitogenome	Analysis
MCZ A‐2853	Bone	1907	“Hung‐ya‐Hsien” [=Hongya, Meishan], Sichuan	Yangtze	16.35	0.99	1 and 2
NMNH 52409	Muscle	1915	“Yachow” [=Ya'an], Sichuan	Yangtze	2.47	0.82	1 and 2
BMNH 1909.7.22.1	Muscle	1909	“Yachow” [=Ya'an], Sichuan	Yangtze	11.22	0.98	1 and 2
BMNH 1945.11.7.1	Liver	1920	Hong Kong Botanical Gardens [?Guangdong or Guangxi]	Pearl	2.58	0.88	1 and 2
ZMB 24105	Muscle	pre‐1922	Guangdong or Guangxi	Pearl	5.66	0.98	1 and 2
ROM 11036	Muscle/Liver	1992	Huangshan, Anhui	Yangtze or SW rivers	2.35	0.82	1 and 2
ROM 11037	Muscle/Liver	1992	Huangshan, Anhui	Yangtze or SW rivers	2.8	0.89	1 and 2
ROM 11038	Muscle/Liver	1992	Huangshan, Anhui	Yangtze or SW rivers	23.97	0.99	1 and 2
ROM 11039	Muscle/Liver	1992	Huangshan, Anhui	Yangtze or SW rivers	5.97	0.97	1 and 2
ROM 11041	Muscle/Liver	1992	Xi'an, Shaanxi	Yellow	3.52	0.94	2
ROM 11045	Muscle/Liver	1992	Zhangjiajie (Dayong), Hunan	Yangtze	8.43	0.98	2
ROM 11047	Muscle/Liver	1992	Chongqing	Yangtze	8.87	0.93	2
ROM 11048	Muscle/Liver	1992	Chongqing	Yangtze	4.38	0.93	2
ROM 11052	Muscle/Liver	1992	Yuanqu, Shanxi	Yellow	66.88	0.99	2
ROM 11053	Muscle/Liver	1992	Yuanqu, Shanxi	Yellow	6.97	0.97	2
ROM 11054	Muscle/Liver	1992	Unknown	Pearl	4.35	0.95	2
ROM 11055	Muscle/Liver	1992	Unknown	Pearl	3.94	0.95	2

Abbreviations: BMNH, Natural History Museum, London; MCZ, Museum of Comparative Zoology, Harvard University; NMNH, Smithsonian National Museum of Natural History; ROM, Royal Ontario Museum, Toronto; ZMB, Museum für Naturkunde, Berlin.

To reconstruct the evolutionary history of allopatric giant salamander populations across China, and to clarify the phylogenetic relationships of these populations, we analyzed a series of historical museum specimens using Next‐Generation Sequencing methods. Due to the estimated size of the cryptobranchid genome (~50 Gb; Morescalchi, 1975), the absence of a reference genome, and the likely degraded/fragmented condition of genetic material in historical samples, our analyses use mitochondrial DNA (mtDNA). Our findings establish a new mitogenomic framework for understanding cryptobranchid diversity and diversification in the context of China's geological history and provide a new taxonomic assessment of Chinese giant salamanders to inform conservation management of the world's largest amphibians.

## 2 METHODS

### 2.1 DNA extraction, library amplification and sequencing

Samples consisted of liver, muscle, or bone, with historical soft tissue preservation including both formalin fixation and suspension in ethanol (Table 1). All extractions and DNA library builds were performed in a dedicated ancient DNA laboratory within the Natural History Museum, London, which is physically isolated from post‐PCR laboratories. All surfaces were presterilized with bleach solution, and all instruments, plastics, and reagents were UV‐irradiated prior to use where appropriate. An ancient DNA protocol was employed to optimize recovery of phylogenetically informative sequences of less than 50 bp from degraded material (Dabney et al., 2013). For soft tissue samples, up to 25 mg of starting material was cut and lysed overnight, with agitation, in 180 μl ATL Buffer and 20 μl proteinase K at 56℃. For bone samples, material was ground to a fine dust using a pestle and mortar, and 50 mg of the sample was subjected to an 18‐hr digestion stage, with agitation, in a 0.5 M EDTA, 10 mg/ml proteinase K solution at 37℃ to decalcify and lyse cells. Postdigestion, the phenol–chloroform, silica spin column protocol described in Dabney et al. (2013) was followed for DNA extraction for all tissue types. Double‐stranded library builds with double‐index inserts to minimize risk of “bleed‐through” during Next‐Generation sequencing were conducted following the protocol of Meyer and Kircher (2010). Amplified libraries were shotgun sequenced on a single lane, using a mid‐output kit, on an Illumina NextSeq500 platform.

Adapter sequences were trimmed and overlapping paired‐end reads were merged using AdapterRemoval (Lindgreen, 2012). Reads were mapped to a consensus sequence of previously published full‐length Chinese giant salamander mitogenomes (GenBank accession numbers: KX268733.1, NC_004926.1, AJ493192.1) using BWA (Li & Durbin, 2009). Parameters were optimized for both ancient DNA and Illumina platform‐specific sequencing error as in Shubert et al. (2012). The “seed” option was disabled and mismatch penalty (−*n*) was set to allow a 2% uniform (0.04) base error rate. Read mapping was initially performed using a quality score threshold of q30 (probability [*p*] of a base being called correctly = 99.9%); however, this resulted in low‐coverage depth in some regions of the mitogenome for some samples, and so reads were remapped using a quality score of q20 (*p* = 99%). To ensure this did not allow inclusion of incorrect base calls, consensus sequences generated from both q30 and q20 mapping were compared for each individual; no differences were observed between sequences and coverage was marginally increased for reads mapped with q20, so this parameter was applied to map reads for all samples. Mapping statistics were calculated using SAMtools (Li et al., 2009), BCFtools, and BEDtools (Quinlan & Hall, 2010) suites, and x‐fold coverage was obtained using Qualimap (Okonechnikov, Conesa, & García‐Alcalde, 2015). Sequences were included in analyses if they had an x‐fold coverage value of >2, with ≥80% of the mitogenome covered at ≥2 read depth, and with read coverage at key coding genes targeted in analyses.

### 2.2 Phylogenetic analyses

We conducted phylogenetic analyses using two different subsets of mitogenomic data. First, we only included sequence data for (a) the five pre‐1922 museum samples (comprising samples from the Pearl drainage/Nanling mountains, and the northern upper Yangtze drainage in Sichuan) which were collected before large‐scale translocations of giant salamanders took place across China; and (b) four 1992 samples from Huangshan, which have previously been identified as a distinct clade, and are from a geographic region not represented by older museum collections and which was economically undeveloped and considered unlikely to have been affected by translocations in the 1990s before development of large‐scale salamander farming (Murphy et al., 2000). Our second analysis also included sequence data from eight more 1992 samples, which reportedly originated from additional localities across the Yangtze, Yellow, and Pearl drainages (Table 1; Murphy et al., 2000). Neighbor‐Joining and Bayesian tree topologies in both analyses were fully congruent, with very similar support values, and so only Bayesian phylogenies are reported here (see also Text S2 and Figure [Link], [Link]).

Multiple alignments were prepared using Muscle (Edgar, 2004) on the GeneiousPro platform (Kearse et al., 2012) for concatenated rRNAs, concatenated tRNAs, and 13 protein‐coding genes. Annotations were determined by MITOS (Bernt et al., 2013) and with reference to previously published and annotated Chinese giant salamander sequences on GenBank. Alignments were concatenated using BioPython (Cock et al., 2009). One whole Japanese giant salamander mitogenome sequence (GenBank accession number: AB208679.1) and one whole hellbender mitogenome sequence (GenBank accession number: GQ368662.1) were used as outgroup taxa. Molecular phylogenies were constructed using both Neighbor‐Joining and Bayesian MCMC inference. The Neighbor‐Joining tree was constructed using MEGA v7.0.18 (Kumar, Stecher, & Tamura, 2016) and the Kimura 2‐parameter model, which included transitions and transversions with G set to 0.68. The Bayesian phylogeny was generated in MrBayes (Huelsenbeck & Ronquist, 2001) via the Cipres Science Portal (Miller, Pfeiffer, & Schwartz, 2010). The best‐fitting model of nucleotide substitution was selected for each partition using jModelTest2 (Table S6; Darriba, Taboada, Doalla, & Posada, 2012), running two independent chains for 2,000,000 generations each, sampling trees and model parameters from the posterior every 2,000 iterations, and discarding the first 25% of each run as burn‐in. Postrun statistics and Are We There Yet (AWTY: Wilgenbusch, Warren, & Swofford, 2004) were used to check for chain convergence and sufficient sampling, before creating a 50% consensus tree. All trees were visualized in FigTree v.1.3.1 (Rambaut & Drummond, 2010).

A Bayesian phylogeny and minimum‐joining network were generated to test whether our data agreed with the recent phylogenetic findings of Yan, Lü, et al. (2018) based on mitochondrial gene data (partial cyt *b*, *COI*, D‐loop). The D‐loop was omitted due to low read coverage, and *COI* gene data had many ambiguous base calls in some specimens; a partial cyt *b* (1,029 bp) dataset was therefore generated for 88 cryptobranchoid taxa (Table S7) using our newly generated data combined with data from Yan, Lü, et al. (2018). A phylogenetic tree was constructed in MrBayes (Huelsenbeck & Ronquist, 2001) using a GTR + G model of nucleotide substitution. Two independent chains were run for 5,000,000 generations each, sampling trees and model parameters from the posterior every 5,000 iterations and discarding the first 25% of each run as burn‐in, to generate a 50% consensus tree. A median‐joining network was generated and edited in PopART using default parameters (Leigh & Bryant, 2015).

### 2.3 Divergence dating and species delimitation

Divergence dates between the three geographically distinct Chinese giant salamander clades were estimated by creating a time‐calibrated species tree for the Cryptobranchoidei Noble, 1931 (Cryptobranchidae Fitzinger, 1826 + Hynobiidae Cope, 1859) using BEAST v.1.8.4 (Drummond, Suchard, Dong, & Rambaut, 2012). The Cryptobranchoidei species‐level tree contained 80 complete or partial mitogenomes, including 12 sequences for the three currently recognized cryptobranchid species (the five pre‐1922 museum samples, the four 1992 samples from Huangshan, two *A. japonicus* sequences, and one *C*. *alleganiensis* sequence), and 68 sequences from 37 hynobiid species recognized on http://www.amphibiaweb.org (Table S7). The same coding regions and partitions as described above were applied, and Jmodeltest2 (Darriba et al., 2012) was used to determine the appropriate nucleotide substitution model (Table S8).

A time‐calibrated tree was generated using fossil, geological, and molecular data. The ingroup node (representing the divergence date of Hynobiidae and Cryptobranchidae) was constrained using the oldest known member of crown group Cryptobranchidae, the pancryptobranchan *Chunerpeton tianyiense* (Gao & Shubin, 2003) from the Bathonian–Oxfordian (Mid to Upper Jurrasic; Marjanović & Laurin, 2014); mean stage ages were used, with lower and upper bounds representing start of the Bathonian and end of the Oxfordian (162.8 ± 5.5 Mya). The age of the Cryptobranchidae node was calibrated using the oldest known Eurasian Cenozoic cryptobranchid, *Aviturus exsecratus* (Gubin, 1991; Vasilyan & Böhme, 2012), given a minimum node age of 56 Mya following Marjanović & Laurin (2007) and Marjanović and Laurin (2014). Hynobiids have a poor fossil record and known fossil hynobiids are obviously younger than the likely date of the family's origin, so two molecular date ranges were obtained by analyses of 29 nuclear genes in Chen et al. (2015): origin of crown group of extant Hynobiidae (mean age, 135.1 Mya; soft bounds, 120.2–150.3 Mya), and major diversification of extant hynobiids (all hynobiids without *Onychodactylus* spp.; mean age, 40.2 Mya; soft bounds, 34.5–46.2 Mya). The same nucleotide substitution models as used for previous analyses were set for each partition, and an uncorrelated, log‐normal clock was employed with a “speciation: birth–death” tree prior using a random starting tree. The analysis was run for 2×10^8 ^generations, sampling from the prior every 2,000 generations. TRACER v.1.5 (Rambaut, Suchard, Xie, & Drummond, 2014) was used to check for chain convergence and sufficient sampling, before creating an MCC tree in TreeAnnotator v.1.8.4 (Drummond et al., 2012), and analysis was repeated sampling only from the prior to check that results were data‐driven.

General Mixed Yule Coalescent (GMYC) modeling (Pons et al., 2006) in R v.3.4.4 was employed as an additional approach to investigate whether discrete clades of Chinese cryptobranchids could be interpreted as separate species. This method delineates species by finding the Maximum Likelihood solution for a model that searches for locations in a tree where there are transitions in branching patterns from speciation (based on a Yule model) to intra‐species genealogical branching (based on a neutral coalescent model). The time‐calibrated, ultrametric BEAST tree was used as input for this analysis, and the model was run under the single‐threshold scenario.

## 3 RESULTS

### 3.1 Phylogenetic analyses

We analyzed samples from 21 historical Chinese giant salamander museum specimens with collection locality information obtained from China before 1922, and 20 tissue samples previously analyzed by Murphy et al. (2000) and Yan, Lü, et al. (2018) obtained in 1992 from giant salamanders with reported wild localities. Available samples represent four separate river drainages and nine provinces or equivalent administrative units and include the holotypes of *Andrias davidianus* and *Megalobatrachus sligoi* (Table S1). We retrieved partial or near‐complete mitogenomes for 17 samples, representing five historical samples including the holotype of *M. sligoi* (BMNH 1945.11.7.1) and 12 samples from 1992, ranging from 2.36 × to 66.88 × coverage (Table 1). Sequences were translated to amino acid residues, which showed no nonsense base calls. The final alignment was 15,211 bp in length; excluding sites with gaps and missing data, there were 7,208 invariable sites and 62 polymorphic sites, of which 13 were singleton variants and 49 were potentially parsimony informative. Nucleotide diversity (*Pi*) was 0.003, and haplotype diversity (*Hd*) was 0.949 with 12 unique haplotypes. When sites with missing data and gaps were excluded, three sets of specimens (ROM 11036, 11038, 11039; ROM 11052–11054; USNM 52409, MCZ A‐2853) showed identical haplotypes.

**Figure 3:**
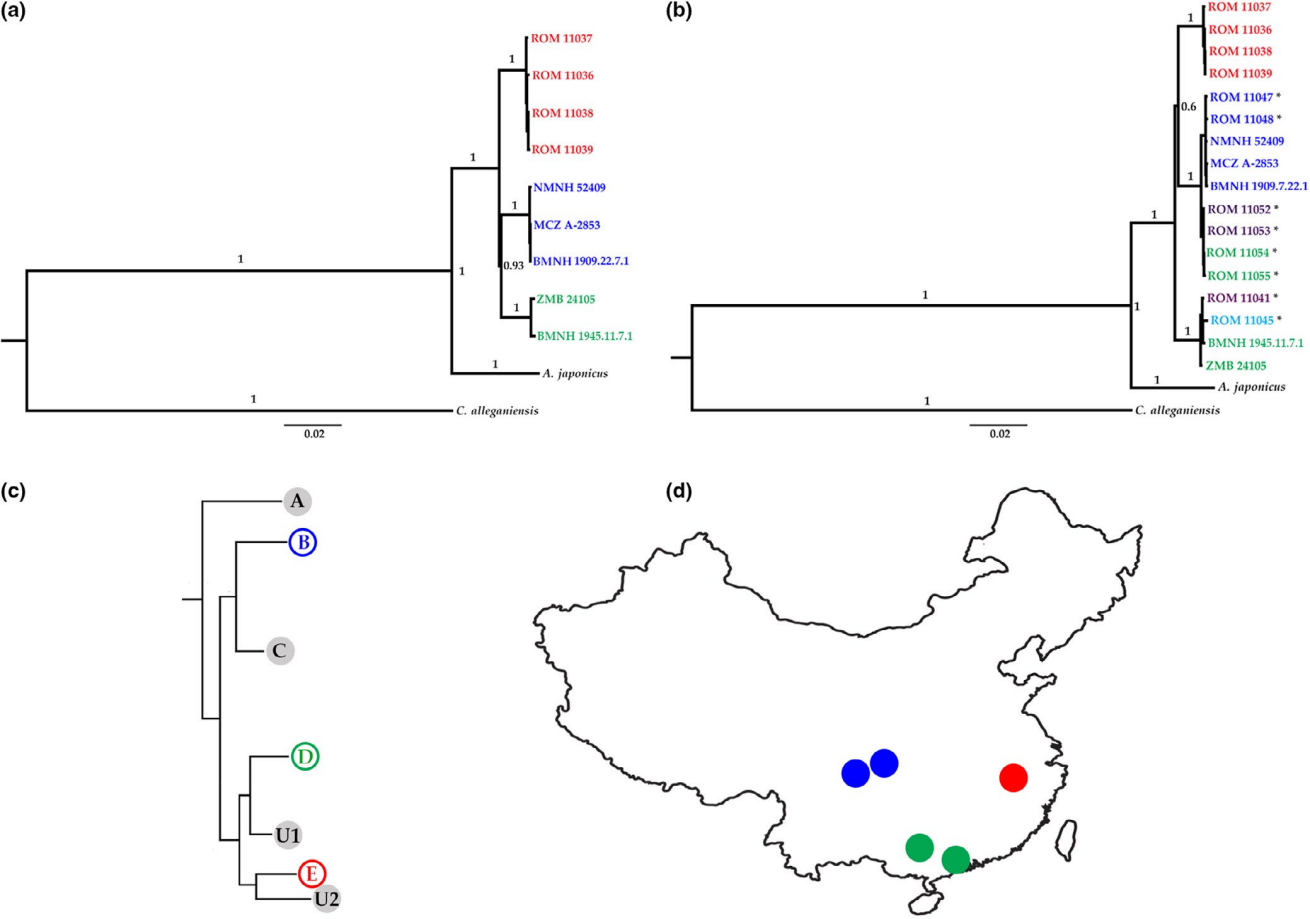
(a, b) Bayesian MCMC phylogenies for Chinese *Andrias* samples. (a) Phylogeny including only pre‐1922 museum samples and 1992 samples from Huangshan. (b) Phylogeny including all partial or near‐complete mitogenomes. Key: red, Huangshan; purple, Yellow River drainage (central Chinese mountain region); dark blue, northern Yangtze River drainage (central Chinese mountain region); pale blue, southern Yangtze River drainage; green, Pearl River drainage/Nanling mountains. Asterisks indicate 1992 samples from localities potentially affected by translocations. (c) Phylogeny of modern Chinese *Andrias* samples from Yan, Lü, et al. (2018) showing five lineages identified from reportedly wild‐caught individuals (a–e), and two lineages identified from farmed individuals (U1, U2); lineages recognized in our study shown in same colors as in Figure 3a. (d) Geographic distribution of samples assigned to different Chinese *Andrias* lineages in Figure 3a (see Figure 1 for more information)

Our first analysis (pre‐1922 museum samples and four 1992 samples from Huangshan) identifies three distinct Chinese giant salamander matrilines: the southwestern (Pearl/Nanling) and northern (Yangtze/Sichuan) samples form sister clades to a southeastern (Huangshan) clade, with complete congruence between phylogenetic placement and geographic location (Figure 3a). Comparison with cyt*b* data indicates these clades correspond to matrilines B, D, and E of Yan, Lü, et al. (2018) (clade B = Yangtze/Sichuan samples: USNM 52409, MCZ A‐2853, BMNH 1909.7.22.1; clade D = Pearl/Nanling samples: ZMB 24105, BMNH 1945.11.7.1; clade E = Huangshan samples: ROM 11036–11039) (Figures S3 and S4). The three groups form a distinct, well‐supported monophyletic clade that is sister to the Japanese giant salamander. All Bayesian posterior support values are >0.9, with all node bipartitions and branch support values at 1, apart from the node separating the Pearl/Nanling and Yangtze/Sichuan clades (support value = 0.93).

Our second analysis (all samples) obtained a different topology, with the Huangshan and northern Yangtze/Sichuan clades forming sister clades to the Pearl/Nanling clade (Figure 3b). Within these three main clades, two 1992 samples from the northern Yangtze drainage in Chongqing (ROM 11047–11048) cluster with the two historical northern Yangtze/Sichuan samples; four 1992 samples (ROM 11052–11053 from Yellow River drainage in Shanxi; ROM 11054–11055 from unknown locality in Pearl River drainage) form a separate subclade that is sister to the northern Yangtze/Sichuan clade; and two 1992 samples (ROM 11041 from Yellow River drainage in Shaanxi; ROM 11045 from southern Yangtze drainage in Hunan) cluster with the two historical Pearl/Nanling samples. All additional 1992 samples fall into clades B and D of Yan, Lü, et al. (2018) (clade B = ROM 11047–11048, 11052–11055; clade D = ROM 11041, 11045) (Figures S3 and S4). All posterior support values are high, with all node bipartitions and branch support values at 1, apart from the node separating the Huangshan and northern Yangtze + Yellow River clades, which has a low posterior support value of 0.6.

We used the first alignment (containing only pre‐1922 and Huangshan samples) to investigate between‐population divergence. Within‐population sample sizes are too low to calculate standard measures of population divergence such as *F_ST_*, but fixed nucleotide differences demonstrate clear subdivision into three distinct clades: 76 fixed differences between Yangtze/Sichuan and Pearl/Nanling clades (mean number of nucleotide differences, *k* = 56.20), 68 fixed differences between Yangtze/Sichuan and Huangshan clades (*k* = 40.48), and 53 fixed differences between Pearl/Nanling and Huangshan clades (*k* = 37.53). A pairwise distance matrix of *p*‐distances indicates that genetic distances between individuals are generally very small across the protein‐coding regions of the mitogenome used in this study (Table 2).

**Table 2:** Pairwise distance matrix between specimens used in Bayesian analyses. Distances shown in bottom left, standard errors shown in top right

ID	ROM 11036	ROM 11037	ROM 11038	ROM 11039	ROM 11041	ROM 11045	ROM 11047	ROM 11048	ROM 11052	ROM 11053	ROM 11054	ROM 11055	ZMB 24105	NMNH 52409	MCZ A2853	BMNH 1909.7.22.1	BMNH 1945.11.7.1	*Andrias japonicus*	*Cryptobranchus alleganiensis*
11036	—	0.000	0.000	0.000	0.001	0.001	0.001	0.001	0.001	0.001	0.001	0.001	0.001	0.001	0.001	0.001	0.001	0.002	0.004
11037	0.000	—	0.000	0.000	0.001	0.001	0.001	0.001	0.001	0.001	0.001	0.001	0.001	0.001	0.001	0.001	0.001	0.002	0.004
11038	0.000	0.000	—	0.000	0.001	0.001	0.001	0.001	0.001	0.001	0.001	0.001	0.001	0.001	0.001	0.001	0.001	0.002	0.004
11039	0.000	0.000	0.000	—	0.001	0.001	0.001	0.001	0.001	0.001	0.001	0.001	0.001	0.001	0.001	0.001	0.001	0.002	0.004
11041	0.004	0.004	0.004	0.004	—	0.000	0.001	0.001	0.001	0.001	0.001	0.001	0.000	0.001	0.001	0.001	0.001	0.002	0.004
11045	0.004	0.004	0.004	0.004	0.000	—	0.001	0.001	0.001	0.001	0.001	0.001	0.000	0.001	0.001	0.001	0.001	0.002	0.005
11047	0.006	0.006	0.006	0.006	0.003	0.003	—	0.000	0.000	0.000	0.000	0.000	0.001	0.000	0.000	0.000	0.000	0.002	0.004
11048	0.006	0.005	0.006	0.006	0.003	0.003	0.000	—	0.000	0.000	0.000	0.000	0.001	0.000	0.000	0.000	0.000	0.002	0.004
11052	0.006	0.006	0.006	0.006	0.004	0.004	0.001	0.001	—	0.000	0.000	0.000	0.001	0.000	0.000	0.000	0.000	0.002	0.004
11053	0.006	0.006	0.006	0.006	0.004	0.004	0.001	0.001	0.000	—	0.000	0.000	0.001	0.000	0.000	0.000	0.000	0.002	0.004
11054	0.006	0.006	0.006	0.006	0.004	0.004	0.001	0.001	0.000	0.000	—	0.000	0.001	0.000	0.000	0.000	0.000	0.002	0.004
11055	0.006	0.006	0.006	0.006	0.003	0.004	0.001	0.001	0.000	0.000	0.000	—	0.001	0.000	0.000	0.000	0.000	0.002	0.004
24105	0.004	0.004	0.004	0.004	0.001	0.001	0.003	0.003	0.003	0.003	0.003	0.003	—	0.001	0.001	0.001	0.001	0.002	0.004
52409	0.005	0.005	0.005	0.005	0.003	0.003	0.000	0.000	0.001	0.001	0.001	0.001	0.003	—	0.000	0.000	0.000	0.002	0.004
A2853	0.005	0.005	0.005	0.005	0.003	0.003	0.000	0.000	0.001	0.001	0.001	0.001	0.003	0.000	—	0.000	0.000	0.002	0.004
1909.7.22.1	0.006	0.005	0.006	0.006	0.003	0.003	0.000	0.000	0.001	0.001	0.001	0.001	0.003	0.000	0.000	—	0.000	0.002	0.005
1945.11.7.1	0.005	0.004	0.005	0.005	0.001	0.001	0.004	0.004	0.004	0.004	0.004	0.004	0.002	0.004	0.004	0.004	—	0.002	0.004
*Andrias japonicus*	0.042	0.042	0.042	0.042	0.041	0.042	0.042	0.042	0.042	0.042	0.042	0.043	0.042	0.042	0.042	0.042	0.042	—	0.004
*Cryptobranchus alleganiensis*	0.159	0.159	0.159	0.159	0.159	0.159	0.159	0.159	0.159	0.159	0.159	0.159	0.159	0.159	0.159	0.159	0.159	0.163	—

### 3.2 Divergence dating and species delimitation

**Figure 4:**
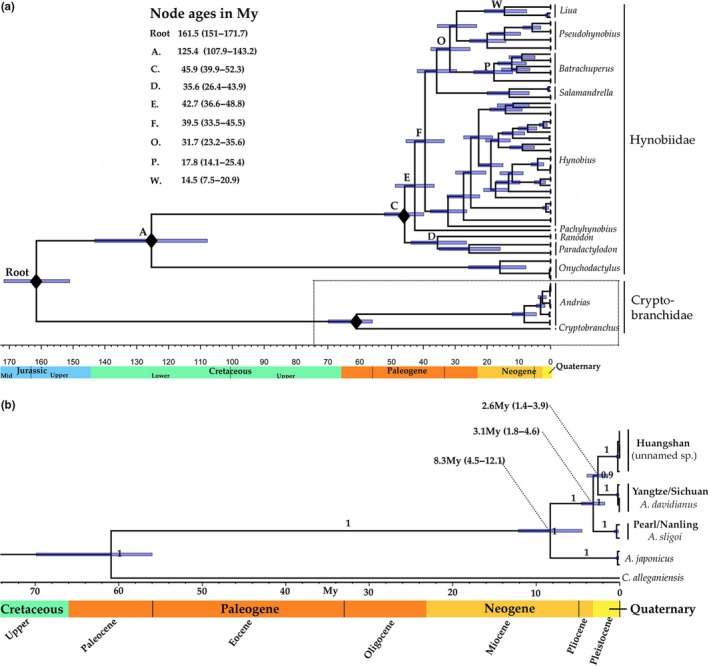
Time‐calibrated BEAST phylogenies. (a) Cryptobranchoidei. (b) Cryptobranchidae. Diamonds indicate calibrated nodes. Node letters correspond to nodes in Chen et al. (2015)

All parameters from BEAST divergence dating runs have effective sample sizes (ESS) of > 200, with convergence reached after 2×10^8 ^generations. The time‐calibrated tree (based on analysis of the subset of samples from populations unlikely to have been affected by translocations) is well‐supported at all taxonomic levels (Figure 4a; Figure [Link], [Link]) and identifies a hynobiid phylogeny largely congruent with recently published tree topologies (Chen et al., 2015; Zhang et al., 2006; Zheng, Peng, Kuro‐o, & Zeng, 2011), indicating good performance of our dataset.

**Figure 5:**
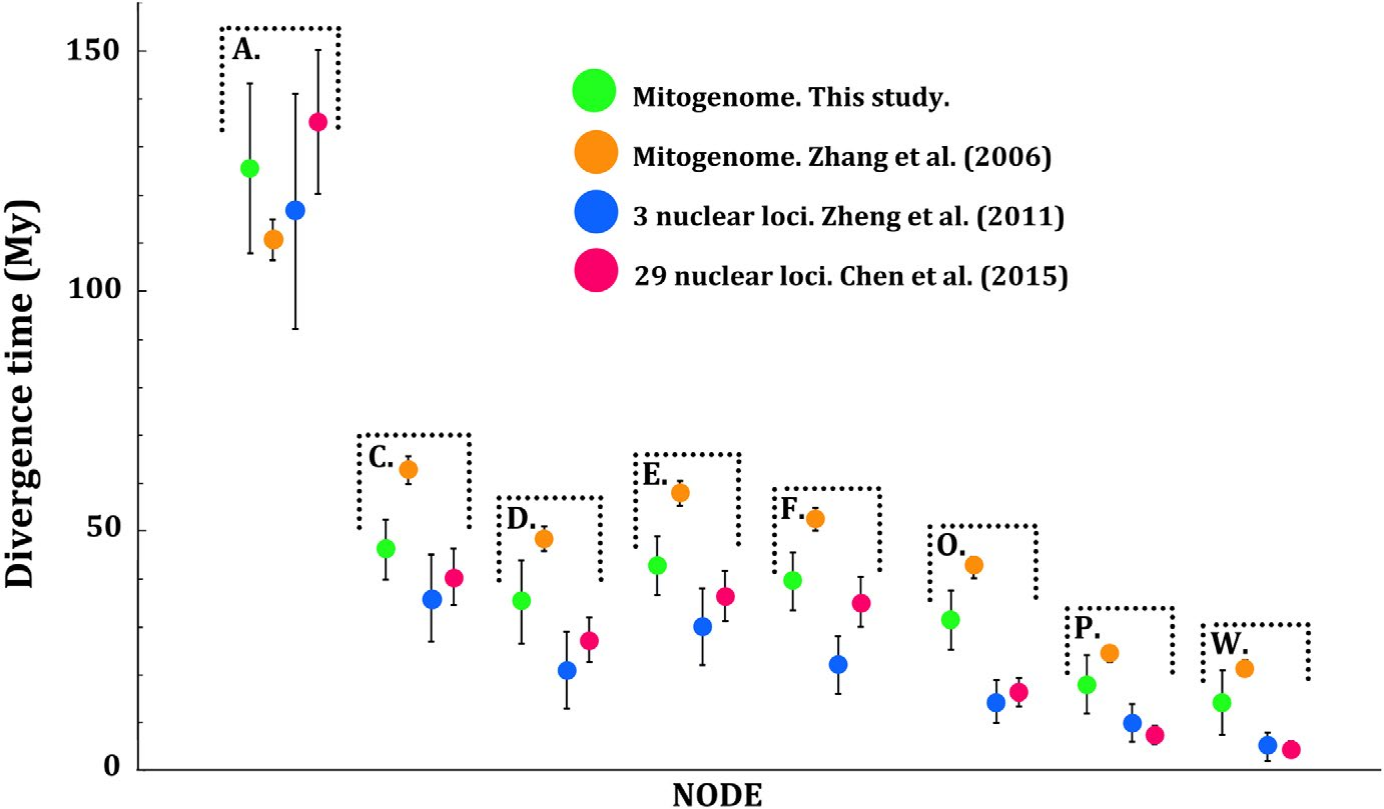
Comparison of divergence date estimates obtained in this study with node age estimates in previous phylogenetic studies of the Hynobiidae (modified from Chen et al., 2015)

Within the Chinese cryptobranchid evolutionary radiation in the time‐calibrated tree, initial divergence of the Pearl/Nanling clade followed by divergence between Yangtze/Sichuan and Huangshan clades is supported with a posterior value of 1 (Figure 4b). Mean node age for divergence between the Japanese giant salamander and the Chinese cryptobranchid radiation is estimated as 8.3 Mya (95% Highest Probability Density [HPD], 4.5–12.1 Mya) (Figures 4 and 5; Table S2). We tested whether divergence between Chinese and Japanese salamanders represents a vicariance event associated with tectonic separation of Japan from mainland Asia, which occurred ~16 Mya (Isozaki, Aoki, Nakama, & Yanai, 2010), but fixing the node age of this split to 16 Mya (*SD* ± 2) in BEAST prevented the run from converging and ESS values of prior and posterior nodes with the Cryptobranchidae did not reach >200 after 2 × 10^8 ^generations, suggesting that the speciation signal in our data is incompatible with this node constraint. Age of this split did not affect node age estimates within the Chinese radiation and so the constraint on this node was removed. Within the Chinese radiation, initial divergence of the Pearl/Nanling clade is estimated at 3.1 Mya (95% HPD, 1.8–4.6 Mya), with subsequent divergence of Yangtze/Sichuan and Huangshan clades 2.6 Mya (95% HPD, 1.4–3.9 Mya).

A single‐threshold GMYC model provided a better fit to the ultrametric, partial mitogenome BEAST tree (containing the five pre‐1922 museum samples and the four 1992 samples from Huangshan) than a null model assuming all Chinese samples represent a single species (likelihood‐ratio test, *p* < 0.001; Text S3 and S4, Figure [Link], [Link]). This model delimited a total of 48 species (maximum likelihood entities) within Cryptobranchoidei, which comprise 28 clusters, indicating that some species identified by the model were represented by a single sequence (Tables [Link], [Link], [Link], [Link]). Four separate Chinese giant salamander species were identified by the model, representing the Huangshan clade (ROM 11036–11039), the Yangtze/Sichuan clade (USNM 52409, MCZ A‐2853, BMNH 1909.7.22.1), and with the Pearl/Nanling clade identified as comprising two separate species (species 1: ZMB 24105; species 2: BMNH 1945.11.7.1). Species delimitation for all species groups had branch support values of 1.

## 4 DISCUSSION

We report the first whole‐mitogenome data for Chinese giant salamanders, a global conservation priority now interpreted as a potential cryptic species complex (Yan, Lü, et al., 2018). In particular, we report the first sequence data from historical specimens, which provide unique insights into the evolutionary history of allopatric populations from distinct biogeographic regions across China. Although most historical samples did not yield DNA (Table S1), probably due to formalin fixation of most fluid‐stored specimens, we successfully recovered mitogenomic data for multiple individuals collected >90 years ago from several Chinese biogeographic regions, predating the period when giant salamanders were moved extensively around China, as well as for late twentieth century samples from additional areas where native populations were unlikely to have been affected by translocations. These data establish a novel baseline for reconstructing cryptobranchid evolution in China and enable new assessment of phylogenetic relationships and taxonomic identities for allopatric populations of the world's largest amphibians.

Our phylogenetic analyses of historical specimens unlikely to have been translocated from their source populations identified three giant salamander clades from separate geographic areas in southern, central, and eastern China (Figures 3, 4). Evolutionary differentiation between these clades is spatially congruent with geographic isolation either by river drainages (Pearl, Yangtze, and Fujian‐Zhejiang hills) or by montane ecoregions across this topographically complex region (Nanling, Sichuan, and Huangshan). Given the limited number of sampling localities associated with historical samples that yielded DNA, and the lack of precise locality data to identify the river system from which the Guangdong/Guangxi and Huangshan specimens originate, it is difficult to test between these two competing biogeographic hypotheses. However, amphibian diversity hotspots in China are primarily in montane ecoregions, and speciation in many Chinese amphibian clades is thought to have been driven by orogenesis rather than by palaeodrainage systems (Chen & Bi, 2007; Hu, Li, Xie, & Jiang, 2012; Li et al., 2018; Li, Yu, Rao, & Yang, 2012; Zhou et al., 2017), suggesting vicariance associated with tectonic uplift is more likely to be responsible for diversification within Chinese cryptobranchids. Indeed, our estimated Pliocene–Pleistocene divergence times for allopatric Chinese giant salamander clades are consistent with extensive topographic modification of southern China associated with rapid uplift of the Qinghai‐Tibet Plateau from ~3.6 Mya onwards (Li et al., 2018; Li, Zhou, Zhao, & Zhang, 2015), and our mean estimated divergence time between the Yangtze/Sichuan and Huangshan clades (2.6 Mya) closely matches the orogenesis of Huangshan ~2.5 Mya (Huang, Diffendal, & Yang, 2002). Conversely, the modern fluvial systems of the Yangtze and Pearl rivers were already established by ~23 and ~11 Mya, respectively (Yan, Yao, et al., 2018; Zheng et al., 2013). The close genetic similarity between giant salamanders across China's central mountain region (Chongqing, Sichuan, Shaanxi, Shanxi), which includes both the northern Yangtze and Yellow River drainages (Figures 1 and 4b), provides further evidence for evolutionary differentiation associated with montane ecoregions rather than river drainages. The occurrence of the basal Chinese cryptobranchid clade in the Nanling mountains is consistent with the known role of this southern montane ecoregion as a Quaternary glacial refugium and “biodiversity museum” that preserved many other palaeoendemic Tertiary lineages which became extinct further north in China (López‐Pujol et al., 2011a, 2011b; Tian et al., 2018). Our results also indicate that earlier divergence between Chinese and Japanese giant salamander clades was not associated with initial geotectonic separation of Japan from mainland Asia; instead, this divergence falls within the subsequent 15–5 Mya interval when the emergent Japanese islands were reconnected to mainland Asia by the Korean Peninsula and the Sea of Japan may have become a large lake, providing both terrestrial and freshwater corridors for cryptobranchid migration (Osozawa et al., 2012).

Mitochondrial DNA can bias node age estimates through site saturation driving divergence estimates toward the calibration point of the ingroup (Arbogast, Edwards, Wakeley, Beerli, & Slowinski, 2002; Nei & Kumar, 2000; Zheng et al., 2011). It is therefore possible that the older calibration dates in our analyses have led to overestimation of divergence dates. However, our dates are well within the range of previous estimates for hynobiids, and our dataset performed substantially better than previous whole‐mitogenome divergence date estimation for this amphibian family (Chen et al., 2015; Zhang et al., 2006; Zheng et al., 2011). Divergence of the Chinese giant salamander species complex was estimated as occurring 4.7–10.3 Mya by Yan, Lü, et al. (2018), a late Miocene–early Pliocene age range considerably older than our estimate. Divergence date estimation in Yan, Lü, et al. (2018) was performed with a small dataset of mitochondrial genes, and the problem of bias toward the ingroup root can be exacerbated if mitochondrial DNA datasets are information‐poor due to factors such as substitution saturation (Arbogast et al., 2002). However, we also recognize that our study suffered from limited sampling due to the difficulty of obtaining historical samples and sequencing degraded DNA, and we were unable to include Clade A of Yan, Lü, et al. (2018), which diverged first in their phylogeny but is not represented by available historical museum specimens. Initial diversification of Chinese cryptobranchids might therefore have occurred slightly earlier than we are able to estimate.

Previous investigations into phylogeographic relationships between Chinese giant salamander populations have proposed that different allopatric clades might represent cryptic species (Murphy et al., 2000; Yan, Lü, et al., 2018), supporting earlier suggestions that more than one cryptobranchid species may occur in China (Sowerby, 1925a, 1925b). However, these studies did not test the taxonomic status of recognized clades (e.g., through use of GMYC), or attempt to associate these clades with either historically erected or new taxonomic names. Although we observed low genetic distances between protein‐coding regions across all sampled individuals, in marked contrast to the pattern of high genetic diversity within the Hynobiidae even at within‐population levels (Matsui, 1987; Matsui, Nishikawa, Utsunomiya, & Tanabe, 2006), comparable low diversity is also seen in Japanese giant salamanders, possibly associated with the unusual life‐history traits of large‐bodied cryptobranchids (e.g., delayed sexual maturity, longevity) (Matsui, Tominaga, Liu, & Tanaka‐Ueno, 2008). Conversely, we observed high levels of fixed nucleotide differences between all three allopatric Chinese giant salamander clades, suggesting they have been isolated from each other for significant periods of time during which local adaptation or genetic drift has led to accumulation of fixed mutations.

Our GMYC analysis demonstrates that all three allopatric Chinese giant salamander clades should be interpreted as representing distinct species. This analysis suggests that the two historical samples from the Pearl/Nanling region (ZMB 24105, BMNH 1945.11.7.1) might also each represent separate species. Yan, Lü, et al. (2018) found that matrilines B and C were probably both associated with the Yellow River, supporting the possibility of regional microendemism within the Pearl/Nanling region. However, precise source population locality data are unavailable for these two historical specimens, so we cannot conclude they were necessarily divergent within the same ecoregion, and both specimens represent clade D of Yan, Lü, et al. (2018). Of these, BMNH 1945.11.7.1 is a low‐coverage sample (2.58×) with 12% of its mitogenome coded as missing, which could generate an artefactual pattern of divergence from ZMB 24105 that could be misinterpreted by the GMYC model. We therefore interpret ZMB 24105 and BMNH 1945.11.7.1 as almost certainly conspecific, with three Chinese cryptobranchid species recognized within China on the basis of our data.

The ~ 150‐year‐old holotype of *Andrias davidianus* did not yield DNA, but this specimen was collected from Zhongba, Chongqing (Liu, 1950), close to the collection localities of our two historical samples from Sichuan (Figure 1). The two 1992 samples from Chongqing included in our second phylogenetic analysis also cluster with these samples (Figure 3b). We can therefore confidently assign the species name *Andrias davidianus* (Blanchard, 1871) to the northern Yangtze/Sichuan clade (= clade B of Yan, Lü, et al., 2018; live individual illustrated in Figure 1). The holotype of *Megalobatrachus sligoi* clusters in a separate clade along with the other historical sample from the Pearl/Nanling region, and so we assign the species name *sligoi*, in the new taxonomic combination *Andrias sligoi* (Boulenger, 1924), to the Pearl/Nanling clade (= clade D of Yan, Lü, et al., 2018). We suggest the new vernacular name “South China giant salamander” should be used to refer to this previously forgotten but valid cryptobranchid species. The Huangshan clade (= clade E of Yan, Lü, et al., 2018) represents a third distinct species; no previously established species name exists for this clade, which has been diagnosed through analysis of tissue samples only, and further work is required to identify referable museum specimens or living individuals to permit formal description and naming.

Our analyses also provide a salutary demonstration of the problems associated with investigating evolutionary patterns using animals that have been moved away from their original distribution by human interference, the context for all previous phylogenetic analyses of Chinese giant salamander populations. Our analysis of samples unlikely to have been affected by translocations demonstrates complete congruence between phylogenetic placement and geographic location. However, addition of more recent samples, including those reportedly obtained from major centers of known exploitation and movement of giant salamanders, leads to increasing loss of biogeographic signal due to incongruity between phylogenetic placement and geography, with incorrect placement observed in 1992 specimens reportedly collected from Xi'an, the Pearl drainage, and possibly also northern Hunan (Figure 3b). Either these “misplaced” individuals originated in a region other than where they were collected (local releases/escapes of translocated individuals), or collection information associated with the samples is incorrect; these samples were “collected, donated, or purchased,” so their origin cannot be confirmed in all cases (Murphy et al., 2000). Giant salamanders were being moved commercially between Xi'an and major trading centers in the Pearl drainage by the 1990s, making it unlikely that these reported localities represent the true provenance of geographically misplaced samples that cluster in our northern Yangtze/Sichuan and Pearl/Nanling clades (Cunningham et al., 2016). In addition, captive giant salamanders have regularly been released in northern Hunan in recent decades (Luo, Liu, & Zhang, 2009), although animals from this region might also represent clade D of Yan, Lü, et al. (2018). Given the huge‐scale expansion of giant salamander farming activities across much of China since the 1990s, the challenges of identifying original provenance for recently sampled animals will be substantially greater, and the phylogenetic and biogeographic conclusions of previous studies should be treated with caution. Recent human‐mediated movement of giant salamanders and mixing of salamander lineages within farms is also highly likely to have led to hybridization of different Chinese species in both captive and wild conditions, as has also been observed between Chinese and more distantly related Japanese giant salamanders (Fukumoto, Ushimaru, & Minamoto, 2015). However, patterns and levels of hybridization are currently unknown and will not be detectable using maternally inherited mitochondrial data alone, necessitating additional genetic methods to screen potential hybrid individuals and establish the extent of this additional major conservation problem.

Our study increases the recognized diversity of living cryptobranchids and adds important new evidence of a previously unknown evolutionary radiation of giant salamander species across China. Additional mainland Chinese populations probably represent further undescribed species, probably including other lineages identified by Yan, Lü, et al. (2018) for which historical museum specimens are unavailable, and also the isolated population reportedly present in the headwaters of the Yangtze River in Qinghai Province at an elevation >2,000 m higher than other known populations, which is likely to be ecophenotypically distinct with different patterns of environmental tolerance (Chen, 2011). Chinese giant salamanders have been introduced to Japan (Fukumoto et al., 2015), and further work is required to determine which Chinese species is represented by this introduced population. Intriguingly, unverified reports of giant salamanders are also known from Taiwan (IUCN, 2018) and the Chin Hills of northern Myanmar (Lane, 1934), suggesting that further giant salamander evolutionary diversity might exist more widely across eastern and southeast Asia. The taxonomic identity of the world's largest amphibian species is also now uncertain. The largest reported giant salamander individual is apparently an individual measuring five feet nine inches (~1.8 m) caught in the early 1920s near Guiyang, Guizhou Province (Chang, 1936; Sowerby, 1925a, 1925b). Historical specimens collected in Guizhou did not yield DNA (Table S1). However, recent giant salamander samples collected from Guizhou cluster with clade D in Yan, Lü, et al. (2018), suggesting that *A. sligoi*, and not *Andrias davidianus*, might be the world's largest amphibian.

These findings highlight the importance and value of underused archival resources for providing unique insights into the evolutionary history of human‐modified faunas, and present a new example of species diversity remaining unrecognized in a large‐bodied vertebrate clade of high conservation concern, with serious implications for management (cf. Iglésias et al., 2010; Stewart, 2013). Further research is required to determine the geographic distributions and diagnostic morphological characters of the newly identified Chinese cryptobranchid species. However, studying and even locating surviving wild populations of any of these species will be challenging due to the severe declines experienced by giant salamanders across China (Turvey et al., 2018). We propose that the newly recognized *A. sligoi* should be assessed as Critically Endangered by IUCN on the basis of Criterion A2cde (estimated reduction in population size) (IUCN, 2001). Chinese environmental legislation should now recognize the existence of multiple giant salamander species, which require separate management plans. Movement of giant salamanders around China by the farming industry and hybridization of different species within salamander farms must be restricted, and existing government‐supported giant salamander release programmes must be modified to identify the origin of captive animals and prevent extralimital introductions of different species. Further efforts should be made to identify and protect sites where remnant populations of different Chinese giant salamander species may still occur. However, as the persistence of viable wild populations of any of these species is now uncertain, genetic screening of animals in farms, zoos, and aquaria should be conducted urgently to identify founder individuals for ex situ conservation breeding of each newly recognized species. We hope that this new understanding of species diversity within China's giant cryptobranchid amphibians has arrived in time to support their successful conservation.

## CONFLICT OF INTEREST

None declared.

## AUTHOR CONTRIBUTIONS

S.T.T., I.B., and A.A.C. designed research; M.M.M. and S.B. performed research; S.T.T., R.M.W., E.Z., and B.T. contributed samples; M.M.M. analyzed data; S.T.T., M.M.M., and B.T. wrote the paper.

## Data Availability

DNA sequences: GenBank accessions MK177461 ‐ MK177477.
