# Supplementary figures and images for: Historical museum collections clarify the evolutionary history of cryptic species radiation in the world's largest amphibians
Source: Ecol Evol. 2019 Sep 16;9(18):10070–84. doi: 10.1002/ece3.5257 (PMC6787787; doi:10.1002/ece3.5257)

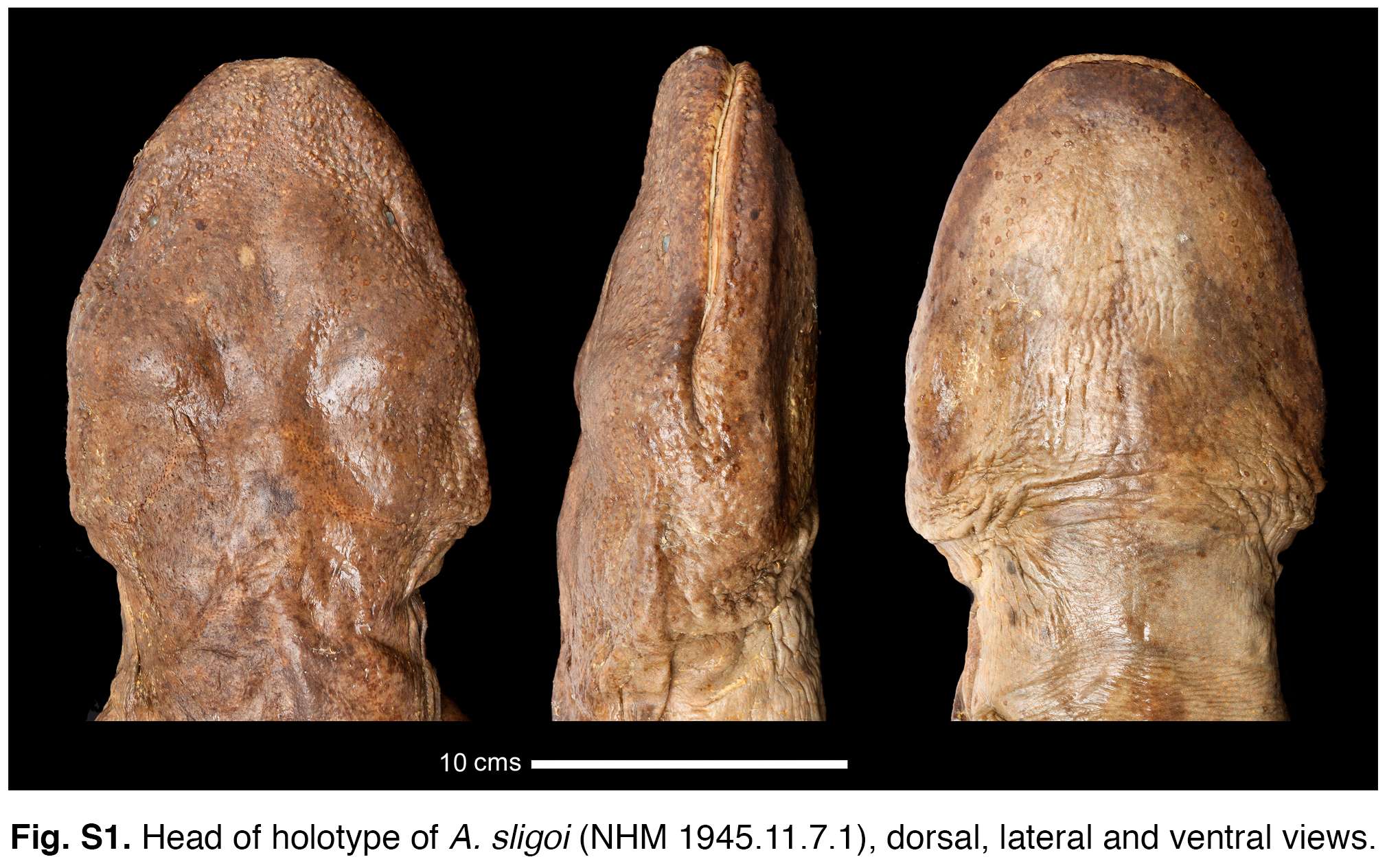

Supplement: Supplementary file 1 [file ECE3-9-10070-s001.tif]

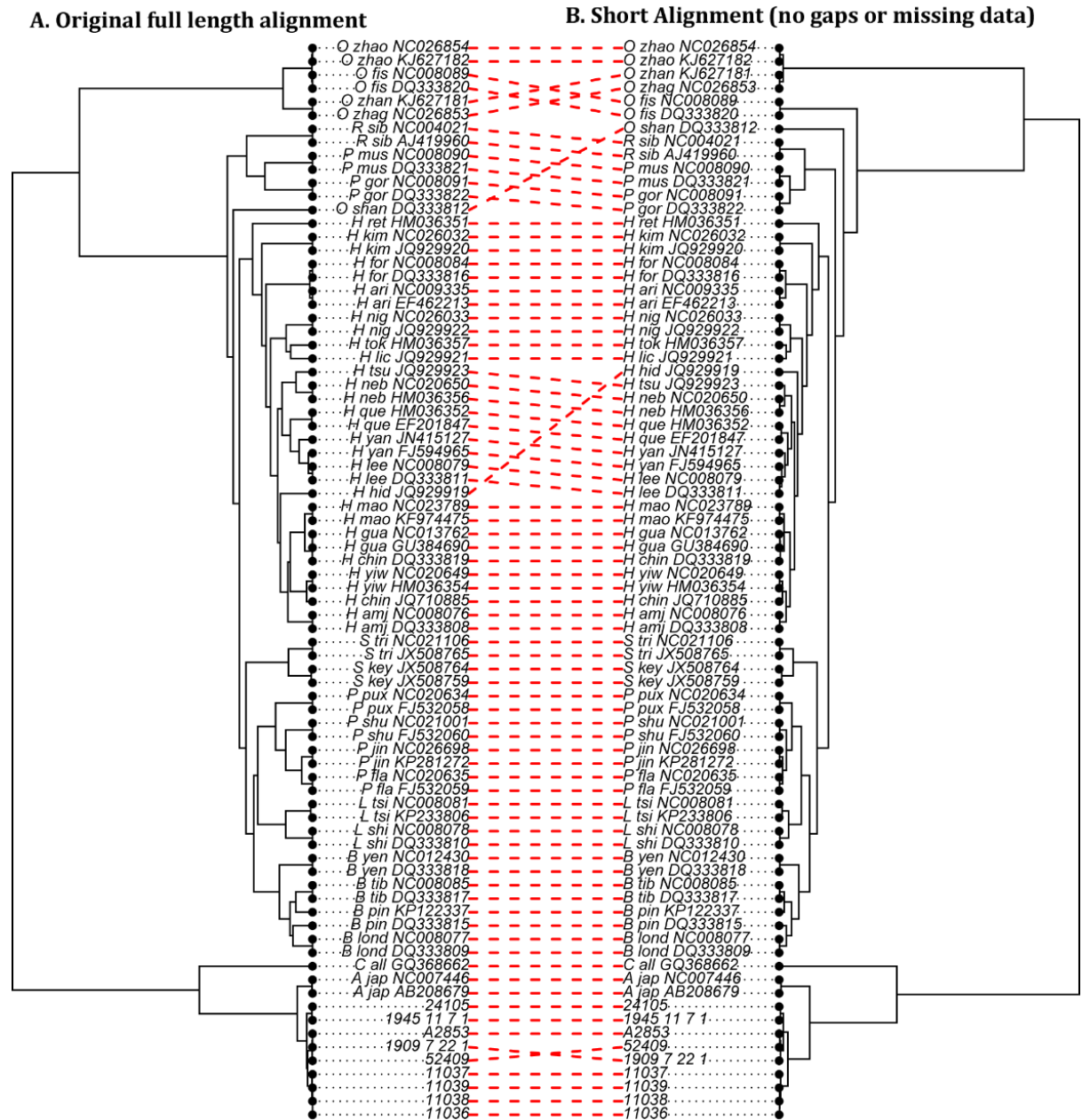

**Fig S6.** Results of the cophylo analysis, showing tip alignment in both trees.

Supplement: Supplementary file 6 [file ECE3-9-10070-s006.pdf]
