# Supplementary material for: Historical museum collections clarify the evolutionary history of cryptic species radiation in the world's largest amphibians
Source: Ecol Evol. 2019 Sep 16;9(18):10070–84. doi: 10.1002/ece3.5257 (PMC6787787; doi:10.1002/ece3.5257)
Supplement: Supplementary file 2 [file ECE3-9-10070-s002.pdf]

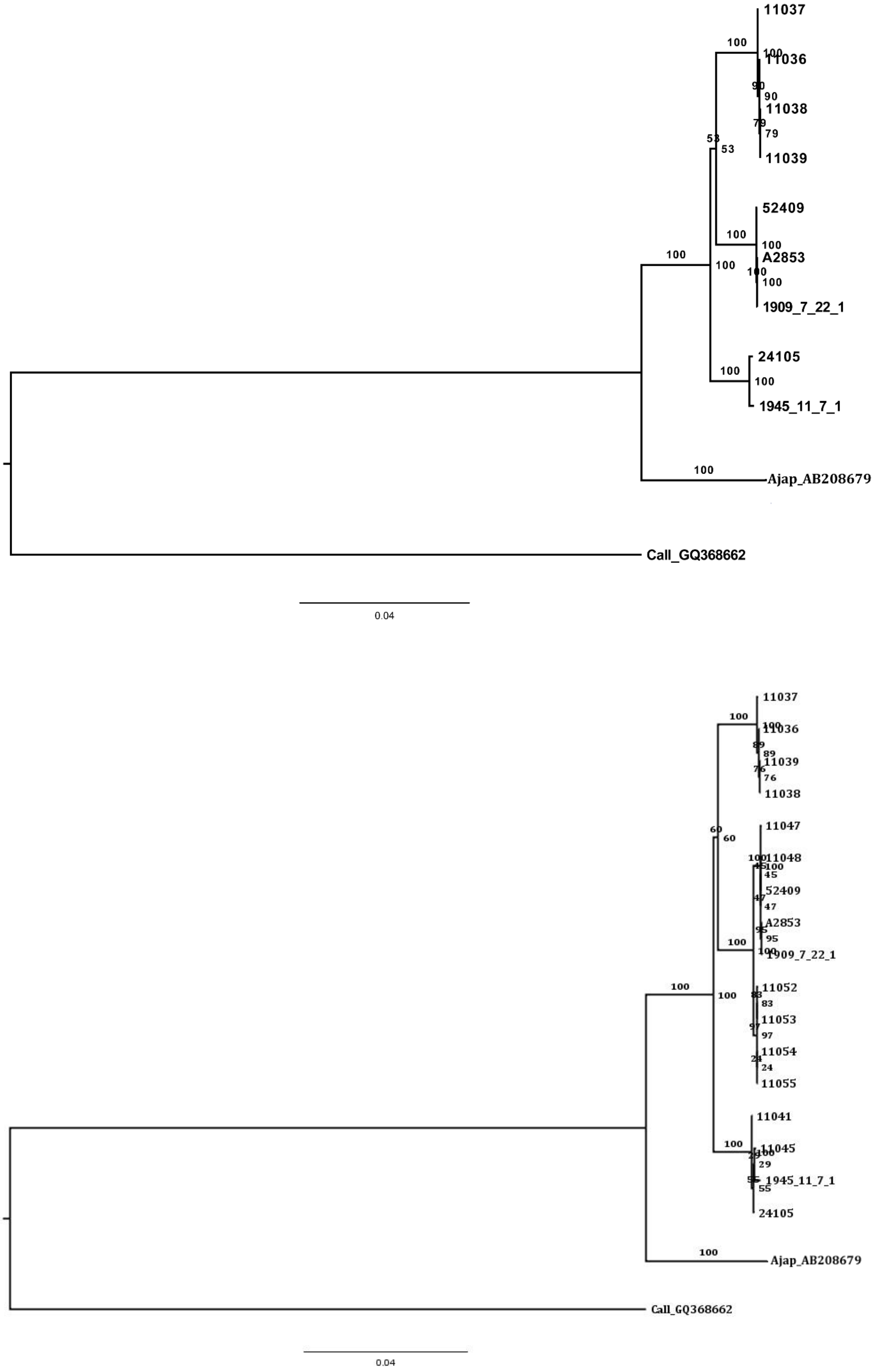

Fig S2. Maximum Likelihood (ML) phylogenies generated in RAxML, for: (top) pre-1922 samples plus four Huangshan samples; (bottom) all samples.
