# Supplementary material for: Historical museum collections clarify the evolutionary history of cryptic species radiation in the world's largest amphibians
Source: Ecol Evol. 2019 Sep 16;9(18):10070–84. doi: 10.1002/ece3.5257 (PMC6787787; doi:10.1002/ece3.5257)
Supplement: Supplementary file 4 [file ECE3-9-10070-s004.pdf]

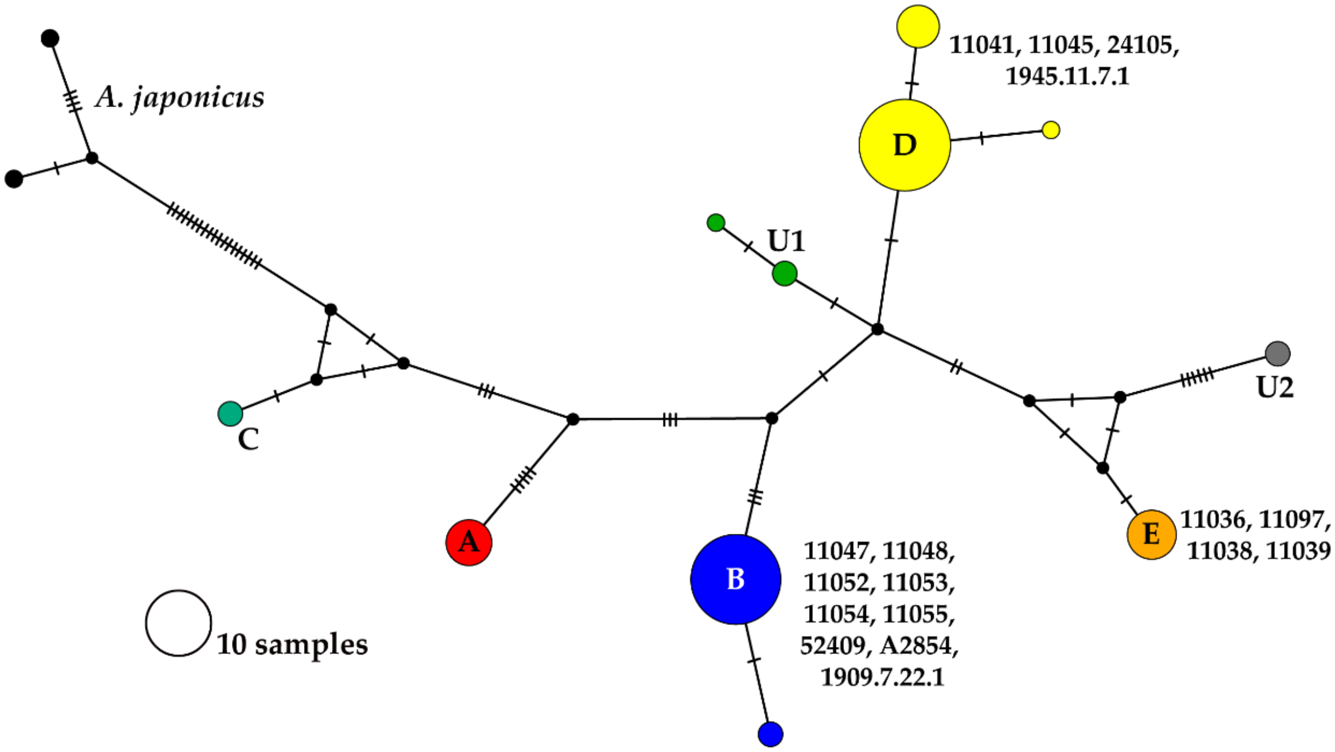

Fig. S4. Median-joining network with concatenated data from this study and Yan et al. (2018a). Specimen IDs show correspondence between samples from this study and clades from Yan et al. (2018a).
