# Supplementary material for: Historical museum collections clarify the evolutionary history of cryptic species radiation in the world's largest amphibians
Source: Ecol Evol. 2019 Sep 16;9(18):10070–84. doi: 10.1002/ece3.5257 (PMC6787787; doi:10.1002/ece3.5257)

Figure S5. Time-calibrated BEAST tree for the Cryptobranchoidei showing node and branch posterior probability support values. All bipartitions at all taxonomic levels are highly supported.

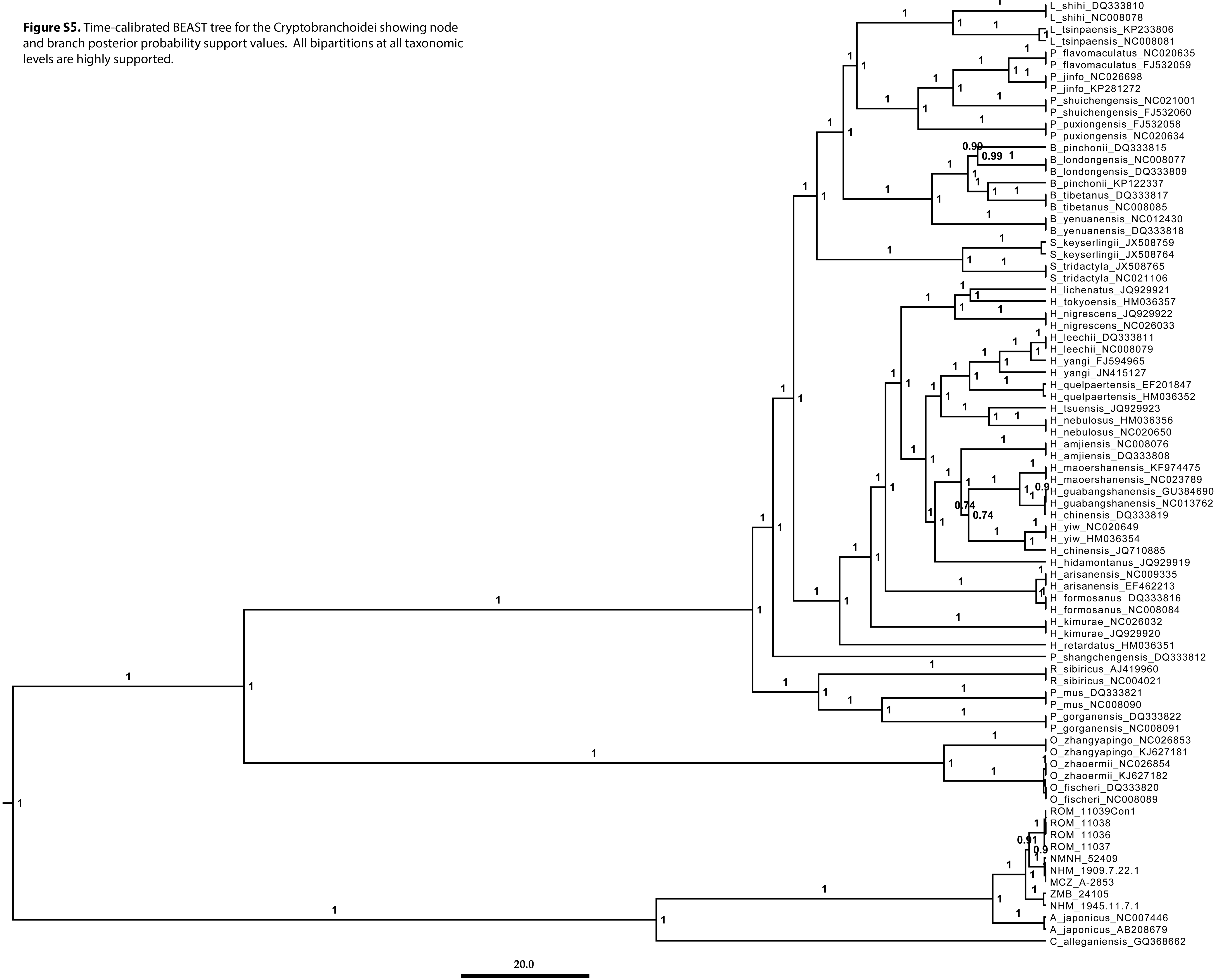

Supplement: Supplementary file 5 [file ECE3-9-10070-s005.pdf]
