# Supplementary material for: Historical museum collections clarify the evolutionary history of cryptic species radiation in the world's largest amphibians
Source: Ecol Evol. 2019 Sep 16;9(18):10070–84. doi: 10.1002/ece3.5257 (PMC6787787; doi:10.1002/ece3.5257)
Supplement: Supplementary file 8 [file ECE3-9-10070-s008.docx]

| **Table S2.** Comparison of shared node divergence date estimates generated in this study, and divergence date estimates generated in previous studies for the Hynobiidae, using whole mitogenome data (Zhang et al. 2006), three nuclear genes (Zheng et al. 2011), and 29 nuclear loci (Chen et al. 2015), respectively. Upper and lower boundaries represent 95% CI. Units in million years (My). Node letters correspond to those in Chen *et al*. (2015), and are indicated in Fig. 4. |
| --- |
|  |
