# Supplementary material for: Historical museum collections clarify the evolutionary history of cryptic species radiation in the world's largest amphibians
Source: Ecol Evol. 2019 Sep 16;9(18):10070–84. doi: 10.1002/ece3.5257 (PMC6787787; doi:10.1002/ece3.5257)
Supplement: Supplementary file 9 [file ECE3-9-10070-s009.docx]

**Table S3.** Species list of all sequences used in the General Mixed Yule Coalescent analyses and the species delimitation suggested by the model.

| **Sequence Number** | **GMYC Species** | **Sequence ID** | **Species** |
| --- | --- | --- | --- |
| 1 | 1 | **ROM 11037** | **“*A. davidianus*” (Huangshan)** |
| 2 | 1 | **ROM 11039** | **“*A. davidianus*” (Huangshan)** |
| 3 | 1 | **ROM 11038** | **“*A. davidianus*” (Huangshan)** |
| 4 | 1 | **ROM 11036** | **“*A. davidianus*” (Huangshan)** |
| 5 | 2 | **NMNH 52409** | **“*A. davidianus*” (Yangtze/Sichuan)** |
| 6 | 2 | **BMNH 1909.7.22.1** | **“*A. davidianus*” (Yangtze/Sichuan)** |
| 7 | 2 | **MCZ A-2853** | **“*A. davidianus*” (Yangtze/Sichuan)** |
| 8 | 3 | NC007446 | *A. japonicus* |
| 9 | 3 | AB208679 | *A. japonicus* |
| 10 | 4 | DQ333821 | *P. musteri* |
| 11 | 4 | NC008090 | *P. musteri* |
| 12 | 5 | DQ333822 | *P. gorganensis* |
| 13 | 5 | NC008091 | *P. gorganensis* |
| 14 | 6 | AJ419960 | *R. sibiricus* |
| 15 | 6 | NC004021 | *R. sibiricus* |
| 16 | 7 | NC009335 | *H. arisanensis* |
| 17 | 7 | EF462213 | *H. arisanensis* |
| 18 | 8 | DQ333816 | *H. formosanus* |
| 19 | 8 | NC008084 | *H. formoanus* |
| 20 | 9 | NC008076 | *H. amjiensis* |
| 21 | 9 | DQ333808 | *H. amjiensus* |
| 22 | 10 | KF974475 | *H. maoershanensis* |
| 23 | 10 | NC023789 | *H. maoershanensis* |
| 24 | 11 | DQ333819 | *H. chinensis* |
| 25 | 11 | GU384690 | *H. guabangshanensis* |
| 26 | 11 | NC013762 | *H. guabangshanensis* |
| 27 | 12 | NC020649 | *H. yiwuensis* |
| 28 | 12 | HM036354 | *H. yiwuensis* |
| 29 | 13 | DQ333811 | *H. leechii* |
| 30 | 13 | NC008079 | *H. leechii* |
| 31 | 14 | HM036356 | *H. nebulosus* |
| 32 | 14 | NC020650 | *H. nebulosus* |
| 33 | 15 | JQ929922 | *H. nigrescens* |
| 34 | 15 | NC026033 | *H. nigrescens* |
| 35 | 16 | NC026032 | *H. kimurae* |
| 36 | 16 | JQ929920 | *H. kimurae* |
| 37 | 17 | JX508765 | *S. tridactyla* |
| 38 | 17 | NC021106 | *S. tridactyla* |
| 39 | 18 | NC008077 | *B. londongensis* |
| 40 | 18 | DQ333809 | *B. londongensis* |
| 41 | 19 | DQ333817 | *B. tibetanus* |
| 42 | 19 | NC008085 | *B. tibetanus* |
| 43 | 20 | NC012430 | *B. yenuanensis* |
| 44 | 20 | DQ333818 | *B. yenuanensis* |
| 45 | 21 | DQ333810 | *L. shihi* |
| 46 | 21 | NC008078 | *L. shihi* |
| 47 | 22 | FJ532058 | *P. puxiongensis* |
| 48 | 22 | NC020634 | *P. puxiongensis* |
| 49 | 23 | NC020635 | *P. flavomaculatus* |
| 50 | 23 | FJ532059 | *P. flacomaculatus* |
| 51 | 24 | NC026698 | *P. jinfo* |
| 52 | 24 | KP281272 | *P. jinfo* |
| 53 | 25 | NC021001 | *P. shuichengensis* |
| 54 | 25 | FJ532060 | *P. shuichengensis* |
| 55 | 26 | NC026853 | *O. zhangyapingo* |
| 56 | 26 | KJ927181 | *O. zhangyapingo* |
| 57 | 27 | NC026854 | *O. zhaoemii* |
| 58 | 27 | KJ627182 | *O. zhaoemii* |
| 59 | 28 | DQ333820 | *O. fischeri* |
| 60 | 28 | NC008089 | *O. fischeri* |
| 61 | 29 | **ZMB 24105** | **“*A. davidianus*” (Pearl/Nanling)** |
| 62 | 30 | **BMNH 1945.11.7.1** | **“*A. davidianus*” (Pearl/Nanling)** |
| 63 | 31 | GQ68662 | *C. alleganiensis* |
| 64 | 32 | DQ333812 | *P. shangchengensis* |
| 65 | 33 | HM036651 | *H. retardatus* |
| 66 | 34 | JQ710885 | *H. chinensis* |
| 67 | 35 | JQ929919 | *H. hidamontanus* |
| 68 | 36 | EF201847 | *H. quelpaertensis* |
| 69 | 37 | HM036352 | *H. quelpaertensis* |
| 70 | 38 | JN415127 | *H. yangi* |
| 71 | 39 | FJ594965 | *H. yangi* |
| 72 | 40 | JQ929923 | *H. tsuensis* |
| 73 | 41 | JQ929921 | *H. lichenatus* |
| 74 | 42 | HM036357 | *H. tokyoensis* |
| 75 | 43 | JX508759 | *S. keyserlingii* |
| 76 | 44 | JX508764 | *S. keyserlingii* |
| 77 | 45 | DQ333815 | *B. pinchonii* |
| 78 | 46 | KP122337 | *B. pinchonii* |
| 79 | 47 | KP233806 | *L. shihi* |
| 80 | 48 | NC008081 | *L. shihi* |
