# Supplementary material for: Historical museum collections clarify the evolutionary history of cryptic species radiation in the world's largest amphibians
Source: Ecol Evol. 2019 Sep 16;9(18):10070–84. doi: 10.1002/ece3.5257 (PMC6787787; doi:10.1002/ece3.5257)
Supplement: Supplementary file 10 [file ECE3-9-10070-s010.docx]

**Table S4.** Node age comparison from Beast runs on two alignments, the original full-length alignment and the second alignment (conserved regions extracted, gaps and missing data removed).

| **Node** | **First Analysis - Original Full Length Alignment** | | | **Second Analysis - Short Alignment** | | |
| --- | --- | --- | --- | --- | --- | --- |
|  | Mean | 95% HPD Lower | 95% HPD Upper | Mean | 95% HPD Lower | 95% HPD Upper |
| **Ingroup** | 161.53 | 151.08 | 171.73 | 161.98 | 151.39 | 172.74 |
| **Hynobiidae_All (Node A)** | 125.39 | 107.85 | 143.17 | 131.92 | 114.26 | 149.26 |
| **Hynobiidae_Crown (Node C)** | 45.86 | 39.9 | 52.23 | 42.02 | 35.51 | 48.67 |
| **Cryptobranchidae** | 60.91 | 56 | 69.87 | 63.38 | 56 | 78.21 |
| ***Andrias*** | 8.32 | 4.52 | 12.19 | 16.4 | 1.93 | 46.98 |
| **Chinese *Andrias*** | 3.17 | 1.79 | 4.57 | 4.75 | 0.45 | 12.07 |
| **Node D** | 35.6 | 36.4 | 43.9 | 13.87 | 1.97 | 28.87 |
| **Node E** | 42.7 | 36.6 | 48.8 | 30.27 | 18.15 | 41.18 |
| **Node F** | 39.5 | 33.5 | 45.5 | 26.55 | 12.62 | 37.77 |
| **Node O** | 31.7 | 23.2 | 35.6 | 16.33 | 3.75 | 26.24 |
| **Node P** | 17.8 | 14.1 | 25.4 | 10.72 | 2.17 | 18.93 |
| **Node W** | 14.5 | 7.5 | 20.9 | 5.01 | 0.47 | 11.14 |
