# Supplementary material for: Historical museum collections clarify the evolutionary history of cryptic species radiation in the world's largest amphibians
Source: Ecol Evol. 2019 Sep 16;9(18):10070–84. doi: 10.1002/ece3.5257 (PMC6787787; doi:10.1002/ece3.5257)
Supplement: Supplementary file 11 [file ECE3-9-10070-s011.docx]

**Table S5.** Comparison of GMYC model results.

|  | **Full Length** | **Short** |
| --- | --- | --- |
| **Likelihood of null model** | 17.0718 | 53.76 |
| **ML of GMYC** | 123.4801 | 61.57 |
| **Likelihood Ratio** | 212.8165 | 15.61 |
| ***p*** | 0*** | 0*** |
| **No of ML Clusters (CI)** | 28 (27-28) | 27 (27-31) |
| **Number of ML entities (CI)** | 48 (48-49) | 51 (45-51) |
