# Supplementary material for: Historical museum collections clarify the evolutionary history of cryptic species radiation in the world's largest amphibians
Source: Ecol Evol. 2019 Sep 16;9(18):10070–84. doi: 10.1002/ece3.5257 (PMC6787787; doi:10.1002/ece3.5257)
Supplement: Supplementary file 12 [file ECE3-9-10070-s012.docx]

| **Table S6.** Models of nucleotide substitution for each partition in the phylogeny of Cryptobranchidae (*Andrias* + *Cryptobranchus*). |
| --- |
|  |
