# Supplementary material for: Historical museum collections clarify the evolutionary history of cryptic species radiation in the world's largest amphibians
Source: Ecol Evol. 2019 Sep 16;9(18):10070–84. doi: 10.1002/ece3.5257 (PMC6787787; doi:10.1002/ece3.5257)
Supplement: Supplementary file 14 [file ECE3-9-10070-s014.docx]

| **Table S8.** Models of nucleotide substitution for each partition in the phylogeny of Cryptobranchoidei. | |
| --- | --- |
|  |  |
