# Supplementary material for: Historical museum collections clarify the evolutionary history of cryptic species radiation in the world's largest amphibians
Source: Ecol Evol. 2019 Sep 16;9(18):10070–84. doi: 10.1002/ece3.5257 (PMC6787787; doi:10.1002/ece3.5257)
Supplement: Supplementary file 15 [file ECE3-9-10070-s015.docx]

**TEXT S1: REDESCRIPTION OF HOLOTYPE OF *ANDRIAS SLIGOI***

**Holotype:** BMNH 1945.11.7.1, formerly II.1.1.1.a (Fig. 2, Supplementary Fig. S1), individual found in Hong Kong Botanical Gardens in 1920 and thought to have been brought from the nearby mainland (Guangdong or Guangxi provinces); received by the Zoological Society of London on 17 March 1923, and died 7 November 1945 (Boulenger 1924; Liu 1950; Zoological Society of London, unpublished records).

**Measurements:** Most morphological data were recorded on the fixed holotype specimen to the nearest 1.0 mm using digital callipers; snout-vent length, total length and tail length were **measured digitally from photographs of the specimen next to a measuring scale using ImageJ** (Abràmoff et al. 2004). Snout-vent length (SVL), measured from tip of snout to posterior edge of vent, 596 mm; total length (TTL), measured from tip of snout to tip of tail, 936 mm; tail length (TAL), measured from posterior edge of vent to tip of tail, 340 mm; tail depth (TAD), greatest height of tail including tail fin, 89 mm; head length (HL), measured from rear of mandible to tip of snout, 184 mm; head width (HW), measured at widest point, 152 mm; greatest diameter of eye (EL), 5 mm; widest diameter of naris (NL), 5 mm; interocular distance (IO), shortest distance between orbital borders, 81 mm; distance from front of eye to nostril (EN), 51 mm; internarial distance (IN), 33 mm; distance between axillae on left-hand side of specimen (AX), 297 mm; length of left forelimb (AL), measured from point of limb insertion to tip of longest finger, 105 mm; length of posterior limb (PL), measured from point of limb insertion to tip of longest toe, 168 mm; vent length (VL), 26 mm; first finger length (FI), measured from base of second finger to tip of first finger, 14 mm; second finger length (FII), measured from base of first finger to tip of second finger, 16 mm; third finger length (FIII), measured from base of second finger to tip of third finger, 14 mm; fourth finger length (FIV), measured from base of third finger to tip of fourth finger, 12 mm; first toe length (TI), measured from base of second toe to tip of first toe, 19 mm; second toe length (TII), measured from base of first toe to tip of second toe, 26 mm; third toe length (TIII), measured from base of second toe to tip of third toe, 33 mm; fourth toe length (TIV), measured from base of third toe to tip of fourth toe, 31 mm; fifth toe length (TV), measured from base of fourth toe to tip of fifth toe, 23 mm. Data from Zoological Society of London (unpublished records): measured 3 feet 1 inch when received in March 1923; measured 3 feet 4½ inches and weighed 9½ lbs in November 1929; measured 3 feet 6½ inches and weighed 13 lbs in 1945.

**Description:**Head large, longer than wide; snout truncate in dorsal view, obtusely protruding in lateral view; naris rounded, positioned close to corner of truncated snout; eyes rounded, dorsolaterally positioned, lacking lids; eye diameter equal to naris diameter; internarial distance subequal to interorbital distance; interorbital space between tip of snout is flattened; large rounded temporal protuberance behind each eye, with edge of mouth anterior to maximum height of this protuberance; indistinct labial fold; gular fold present. Body dorsoventrally compressed; 12 distinct costal folds on left-hand side of specimen, costal folds indistinct on right-hand side of specimen; indistinct vertebral groove; large and distinct dorsolateral folds, starting from immediately behind head and terminating in inguinal region; forelimbs relatively short, prominent dermal fold on posterior surface of forelimb; four short fingers, rounded in cross-section with rounded tips; finger length formula FIV < FIII = FI < FII; posterior edge of FIV with dermal fold which is continuous with dermal fold on arm; interdigital webbing absent; callous present on palmar surface of hands. Hindlimb short with large and prominent dermal fold on posterior surface; toe length formula TI < TV < TII < TIV < TIII; toes TIII, TIV and TV with dermal fringes, fringe on toe TV continuous with fringe running along posterior surface of hindlimb; callous present on plantar surface of feet below TI, TII and TIII. Tail shorter than body (36% of TTL), laterally compressed, rounded in cross-section at base; dorsal tail fin extending from trunk, apex of fin rounded in cross-section when level with trunk and becoming increasingly blade-like towards tail tip; tail tip round in lateral view; vent slit-like; tail shorter than body (36% of TTL). Oral cavity not examined due to fragility of specimen. Skin granular; relatively few tubercles present on dorsal surface of head and body, with exception of dorsal surface of snout; large tubercles present on dorsolateral surfaces of head, very few tubercles present between temporal protuberances and posterior to snout; distinct row of unpaired tubercles running circum-marginally to ventral surface of lower jaw and across gular fold; majority of gular region otherwise free of tubercles; small number of tubercles present on dorsolateral aspects of body; lateral surfaces of tail covered in large granules which increase in number and density toward tail tip; ventral surface of tail and thighs lacking large tubercles; pectoral region markedly granular; single row of distinct unpaired tubercles in crescent orientation around each forelimb. Large midline incision on ventral surface running from pectoral region almost to pelvic girdle, viscera protruding.

**Colour of holotype in preservative:** Majority of dorsal and lateral surfaces of head, body, forelimbs and hindlimbs tan, darker blotches present on trunk; ventral surface greyish tan with several darker brown blotches; distinctive rows of dark tan tubercles on lower jaw, gular fold and in crescent-shaped orientation around forelimbs; glandular skin with orange flecks present on pectoral region and tail.

**Remarks:** Several morphological descriptions of *A. davidianus* and *A. japonicus* (Temminck, 1836) state that a diagnostic character of *A. davidianus* is a line of paired tubercles on the lower jaw (Liu 1950; Browne et al. 2012; Sparreboom 2014; Fei and Ye 2016). However, these paired structures are clearly visible on two *A. japonicus* specimens collected in Hilda District, Japan (BMNH 1902.5.19.7, 1902.5.19.8) and absent from a captive-bred Chinese species of giant salamander (BMNH 2005.2263). The reliability of this character to differentiate between *Andrias* species requires further study.

**Distribution and conservation status:** The holotype of *Andrias sligoi* is thought to have originated in Guangxi or Guangdong Province, although precise locality data are unavailable. Individuals assigned to clade D in Yan et al. (2018), which group with the holotype of *Andrias sligoi* in this study (see main text), are also reported from several sites across Guizhou Province, although the wild status of these individuals is not confirmed. Giant salamanders were not detected in recent ecological surveys at 10 sites in Guangxi and one site in Guangdong despite 1.01 years of cumulative search effort, and evidence of electrofishing or the use of poison (known methods for harvesting giant salamanders) were detected at four of these sites (Turvey et al. 2018). Giant salamanders in China are known to be threatened by harvesting for the farming industry (Cunningham et al. 2016), and molecular analysis of farmed giant salamanders demonstrates that *Andrias sligoi* has also been harvested to stock breeding farms (Yan et al. 2018). It is likely that hybridisation with both described and undescribed congeners that have been translocated by the farming industry is a threat to this species; molecular analysis of giant salamanders found recently in the wild in Guangdong demonstrated that these individuals were a different species to *A. sligoi*, and probably represent recent farm escapes/releases (Turvey et al. 2018). Habitat loss and fragmentation are also highly likely to have a detrimental impact on any remaining *A. sligoi* populations; Guangdong and Guangxi are ranked within the top five Chinese provinces for deforestation (Ren et al. 2015), and giant salamanders are known to be associated with environments containing forest cover (Wang et al. 2004). There is an inferred dramatic population decline of giant salamanders in Guangdong and Guangxi; 79% of 336 people living within 1 km of the 11 field survey sites knew of giant salamanders and 107 interview respondents had seen one, but the mean time of the last giant salamander sighting was 24.7 years earlier (Turvey et al. 2018). The species is therefore likely to qualify as Critically Endangered according to IUCN Red List Criteria A1cde.

**TEXT S2: PHYLOGENETIC INFERENCE USING MAXIMUM LIKELIHOOD**

A Maximum Likelihood (ML) method of phylogenetic inference was also used as an alternative method to test robustness of our conclusions. The search for the ML tree was performed using RaxML v8.2.10 (Stamatakis 2014) via the Cipres Science Portal (Miller et al*.* 2010). All parameters were identical to those used in Bayesian Inference, with the number of bootstrap replicates set to 1000. The first alignment contained nine Chinese giant salamander sequences, the Huangshan samples plus pre-1922 samples, in addition to two outgroup taxa; one Japanese giant salamander sequence (AB208679) and one hellbender sequence (GQ368662). The second analyses contained the same two outgroup sequences aligned with all 17 Chinese giant salamander sequences. Results from the ML search were congruent with those of the Bayesian inference with regards to both support values and tree topologies (Supplementary Fig. S2). The exception was that, in contrast to tree 1 in the original analysis, the Pearl River clade diverges first, with high support.

**TEXT S3: RESULTS OF GMYC SPECIES DELIMITATION**

Method: single-threshold
Likelihood of null model: 17.0718
Maximum Likelihood (ML) of GMYC model: 123.4801
Likelihood Ratio (LR): 212.8165
Result of LR Test: 0***

Number of Maximum Likelihood Clusters: 28

Confidence Interval: 27-28

Number of ML entities: 48
Confidence Interval: 48-49

**TEXT S4: ALIGNMENT OF CONSERVED REGIONS**

The original full-length alignment used as a BEAST input was refined by the programme Gblocks (Castresana 2000), which extracts regions of defined sequence conservation to generate a new alignment of conserved regions that carry a more reliable phylogenetic signal. The stringent default parameters of Gblocks were used as some sequences were highly divergent. This new alignment was 14435 bases in length. This alignment was then stripped of all columns containing missing data and ambiguous base calls, which further reduced the alignment to 7789 bases. This dataset was used as input for a Beast run using identical calibration and parameter settings as described in Methods. Tree topology from this analysis was compared with the original BEAST tree (full length alignment) using the ‘all.equal.phylo’ command from the ‘ape’ package (Paradis and Schliep 2018) in R v.3.5.1 (R Core Team 2018). This command performs a global comparison of two phylogenetic trees to determine if they are identical. Placement of each species in the two tree topologies was also compared using the ‘cophylo’ function from the ‘phangorn’ library (Schliep 2011). This function optimally rotates branches in two trees and plots the results to compare tips. The GMYC was optimised over the new topology. The shorter length dataset performed poorly, both in terms of returning an accurate phylogeny and in estimating node ages. After 2×10^8^ generations, effective sample sizes (ESS) were <100 for both the posterior and prior parameters. Analyses in R showed that tree topology differed significantly (Supplementary Fig. S6). Most notably, the *Onychodactylus* clade (within the Hynobiidae) is paraphyletic, while this group is known from previous studies to form a monophyletic group (Chen et al. 2015). At lower taxonomic levels of the phylogeny, many nodes have unreasonably large 95% HPD age ranges, with a trend towards erroneously young mean ages, particularly within the Hynobiidae (Supplementary Table S4). The mean age for the *Andrias* species complex is 16.4 My, which is older than in the original analyses, but with a huge 95% HPD range of 1.93–46.98 My.

The GMYC model performed poorly as a result of being optimised over a tree topology that did not reflect true taxonomic or chronological relationships within the Cryptobranchoidea. This analysis identified a larger number of species (maximum likelihood [ML] entities) than the original analyses (Supplementary Table S5). Within the Chinese giant salamander group, six species were identified by the model: 1 – 11039, 11036, 11038 (Huangshan); 2 – 52409, A2853 (Yangtze/Sichuan); 3 – 11037 (Huangshan); 4 – 1909.7.22.1 (Yangtze/Sichuan); 5 – 1945.11.7.1 (Pearl/Nanling); 6 – 24105 (Pearl/Nanling). We interpret these results as almost certainly representing an overestimation of true species diversity, and we conclude that extracting conserved regions and removing columns with missing data did not improve phylogenetic inference, node age estimates, or identification of species.

**SUPPLEMENTARY REFERENCES**

Abràmoff MD, Magalhães PJ, Ram SJ. 2004. Image processing with ImageJ. *Biophotonics Intern*. 11:36–42.

Browne RK, Li H, Wang Z, Hime PM, McMillan A, Wu M, Diaz R, Hongxing Z, McGinnity D, Briggler JT. 2012. The giant salamanders (Cryptobranchidae): Part A. Palaeontology, phylogeny, genetics, and morphology. *Amphib. Reptile Conserv*. 5:17–29.

Boulenger EG. 1924. On a new giant salamander, living in the Society’s gardens. *Proc. Zool. Soc. London* 1924:173–174.

Castresana J. 2000. Selection of conserved blocks from multiple alignments for their use in phylogenetic analyses. *Mol. Biol. Evol*. 17:540-552.

Cunningham AA, Turvey ST, Zhou F, Meredith HMR, Wei G, Liu X, Sun C, Wang Z, Wu M. 2016. Development of the Chinese giant salamander (*Andrias davidianus*) farming industry in Shaanxi Province, China: conservation threats and opportunities. *Oryx* 50:265–273.

Fei L, Ye C. 2016. Amphibians of China. Volume 1. Beijing: Science Press.

Huang H, Lin B, Guo C, Tang Z, Wu Z. 2014. The complete mitochondrial genome of the *Hynobius maoershanensis* (Caudata, hynobiidae). *Mitochondrial DNA Part A* 27:173–174.

Liu C. 1950. Amphibians of western China. Chicago: Chicago Natural History Museum.

Malyarchuk B, Derenko M, Denisova G. 2013. Phylogeny and genetic history of the Siberian salamander (*Salamandrella keyserlingii*, Dybowski 1870) inferred from complete mitochondrial genomes. *Mol. Phylogent. Evol*. 67:348–357.

Paradis E, Schliep K. 2018. ape 5.0: an environment for modern phylogenetics and evolutionary analyses in R. *Bioinformatics* [doi.org/10.1093/bioinformatics/bty633](https://doi.org/10.1093/bioinformatics/bty633)

Peng R, Zhang P, Xiong J, Gu H, Zeng X, Zou F. 2010, Rediscovery of *Protohynobius puxiongensis* (Caudata: Hynobiidae) and its phylogenetic position based on compete mitochondrial genomes. *Mol. Phylogenet. Evol*. 1:252–258.

R Core Team. 2018. R: a language and environment for statistical computing. R Foundation for Statistical Computing: Vienna, Austria. https://www.R-project.org

Ren G, Young SS, Wang L, Wang W, Long Y, Wu R, Li J, Zhu J, Yu DW. 2015. Effectiveness of China's national forest protection program and nature reserves. *Conserv*. *Biol*. 229:1368–1377.

Schliep KP. 2011. phangorn: phylogenetic analysis in R. *Bioinformatics* 27:592-593.

Sparreboom M. 2014. Salamanders of the old world: the salamanders of Europe, Asia and northern Africa. Zeist, Netherlands: KNNV.

Stamatakis A. 2014. RAxML version 8: a tool for phylogenetic analysis and post-analysis of large phylogenies. *Bioinformatics* 30:1213-1313.

Temminck CJ. 1836. Coup d'oeil sur la fauna des îles de la sonde et de l’empire du Japon. Discours préléminaire destiné à servir d’introduction à la faune du Japon. Amsterdam: Müller.

Turvey ST, Chen S, Tapley B, Wei G, Xie F, Yan F, Yang J, Liang Z, Tian H, Wu M, Okada S, Wang J, Lü J, Zhou F, Papworth SK, Redbond J, Brown T, Che J, Cunningham AA. 2018. Imminent extinction in the wild of the world’s largest amphibian. *Curr. Biol.* 28:R592–594.

Wang X, Zhang K, Wang Z, Ding Y, Wu W, Huang S. 2004. The decline of the Chinese giant salamander *Andrias davidianus* and implications for its conservation. *Oryx* 38:197–202.

Yan F, Lü J, Zhang B, Yuan Z, Zhao H, Huang S, Wei G, Mi X, Zou D, Xu W, Chen S, Wang J, Xie F, Wu M, Xiao H, Liang Z, Jin J, Wu S, Tapley B, Turvey ST, Papenfuss TJ, Cunningham AA, Murphy RW, Zhang Y, Che J. 2018. The Chinese giant salamander exemplifies the hidden extinction of cryptic species. *Curr. Biol.* 28:R590–592.

Zhang P, Chen Y, Zhou H, Liu Y, Wang X, Papenfuss TJ, Wake DB, Qu L. 2006. Phylogeny, evolution and biogeography of Asiatic salamanders (Hynobiidae). *Proc.* *Natl Acad. Sci. U. S. A.* 103:7360–7365.

Zhang P, Chen Y, Zhou H, Wang X, Qu L. 2003. The complete mitochondrial genome of a relic salamander, *Ranodon sibiricus* (Amphibia: Caudata) and implications for amphibian phylogeny. *Mol. Phylogenet. Evol*. 28:620–626.

Zhang P, Wake DB. 2009. Higher-level salamander relationships ad divergence dates inferred from complete mitochondrial genomes. *Mol. Phylogenet. Evol*. 53:492–508.

Zheng Y, Peng R, Kuro OM, Zeng X. 2011. Exploring patterns and extent of bias in estimating divergence times from mitochondrial DNA sequence data in a particular lineage: a case study of salamanders (Order Caudata). *Mol. Biol. Evol*. 28:2521–2535.
